# White Feces Syndrome is closely linked to impaired antioxidant capacity, the aquaculture environment, and microbial dysbiosis in *Litopenaeus vannamei* reared in small-pond culture system

**DOI:** 10.1371/journal.pone.0357891

**Published:** 2026-10-22

**Authors:** Zhuoduo Wang, Xin Zhang, Kangze Xv, Adinda Luthfiah, Yongjie Jiang, Limei Zhao, Beiping Tan, Wei Zhang

**Affiliations:** 1 Guangdong Evergreen Feed industry Co., Ltd, Aquatic Feed Research Institute, Zhanjiang, Guangdong, China; 2 Guangdong Provincial Key Laboratory of Evergreen Aquaculture, Research and Development Department, Zhanjiang, Guangdong, China; 3 Laboratory of Aquatic Animal Nutrition and Feed, College of Fisheries, Guangdong Ocean University, Zhanjiang, Guangdong, China; 4 Key Laboratory of Aquatic, Livestock and Poultry Feed Science and Technology in South China, Ministry of Agriculture, Guangdong Ocean University, College of Fisheries, Zhanjiang, Guangdong, China; 5 Aquatic Animals Precision Nutrition and High-Efficiency Feed Engineering Research Center of Guangdong Province, Guangdong Ocean University, College of Fisheries, Zhanjiang, Guangdong, China; 6 Guangdong Provincial Key Laboratory of Aquatic Animal Disease Control and Healthy Culture, Guangdong Ocean University, College of Fisheries, Zhanjiang, Guangdong, China; Tanta University Faculty of Agriculture, EGYPT

## Abstract

**Background:**

White feces syndrome (WFS) is a major health problem affecting intensive shrimp aquaculture, but its underlying mechanisms remain unclear. Increasing evidence suggests that WFS may involve complex interactions among host physiological status, intestinal microbial dysbiosis, and environmental microbial communities. However, the relationships among these factors within commercial shrimp culture systems remain poorly understood.

**Objectives:**

This study aimed to investigate the associations among intestinal antioxidant capacity, intestinal microbiota, sediment microbial communities, and water quality parameters in *Litopenaeus vannamei* affected by WFS under small-pond culture conditions.

**Methods:**

Healthy and WFS-affected shrimp were collected from a commercial shrimp farm in Zhanjiang, Guangdong Province, China. Intestinal antioxidant parameters, including total antioxidant capacity (T-AOC), superoxide dismutase (SOD), glutathione peroxidase (GSH-Px), and malondialdehyde (MDA), were determined. Intestinal and sediment microbial communities were analyzed using 16S rRNA gene sequencing. Water quality parameters and microbial community composition were compared between healthy and WFS groups.

**Results:**

Compared with healthy shrimp, WFS-affected shrimp exhibited significantly increased intestinal MDA levels and decreased activities of T-AOC, SOD, and GSH-Px, indicating an imbalance in antioxidant status. Significant differences in intestinal microbial community composition were observed between groups, as confirmed by beta-diversity analysis (PERMANOVA, R^2^ = 0.407, P = 0.022). WFS-associated microbial alterations included changes in dominant bacterial taxa and predicted microbial functional potential related to metabolism. The dominant taxa, referring to the most abundant microbial groups based on relative abundance, differed between healthy and WFS-affected shrimp. Sediment microbial communities also showed substantial compositional shifts, suggesting alterations in the surrounding microbial environment. In addition, nitrate and total nitrogen concentrations were significantly reduced in the WFS group.

**Conclusion:**

These findings indicate that WFS occurrence is associated with coordinated alterations in shrimp antioxidant status, intestinal microbial structure, sediment microbial communities, and environmental conditions. This study provides new insights into WFS from a host–microbiota–environment interaction perspective and highlights potential indicators for monitoring shrimp health in aquaculture systems.

## 1. Introduction

Pacific white shrimp (*Litopenaeus vannamei*) is the most widely cultured crustacean species worldwide because of its rapid growth, high feed efficiency, and strong environmental adaptability [1]. The rapid expansion of intensive shrimp farming has substantially increased global production; however, high stocking densities, intensive feeding practices, and environmental deterioration have also increased the frequency of disease outbreaks, posing major challenges to sustainable shrimp aquaculture [2–4].

Among the emerging diseases affecting shrimp culture, White Feces Syndrome (WFS) has attracted considerable attention because of its increasing prevalence and substantial economic impact [5,6]. WFS is characterized by floating white fecal strings, poor feed utilization, growth retardation, and, in severe cases, mortality [5,7]. Unlike classical infectious diseases, WFS is a disease syndrome associated with complex interactions among potential pathogens, host physiological status, intestinal microbial dysbiosis, and environmental conditions. Although several pathogens, including EHP and Vibrio spp., have been reported in WFS outbreaks, their exact contribution and causal relationships remain under debate [4,6,8,9].

Increasing evidence indicates that oxidative stress and intestinal microbial dysbiosis are closely associated with disease susceptibility in aquatic animals. Oxidative stress occurs when the production of reactive oxygen species (ROS) exceeds the capacity of antioxidant defense systems, resulting in disruption of redox homeostasis, intestinal epithelial damage, and impaired immune function [10,11]. Meanwhile, the intestinal microbiota plays fundamental roles in nutrient metabolism, immune regulation, pathogen exclusion, and maintenance of intestinal homeostasis [12–14]. Alterations in microbial community composition may further aggravate oxidative damage and intestinal dysfunction, thereby increasing host susceptibility to disease [15,16].

In shrimp, intestinal microbial dysbiosis has been reported under a variety of pathological and environmental stress conditions, including White Feces Syndrome (WFS), acute hepatopancreatic necrosis disease (AHPND), nitrite exposure, and starvation [17–20]. These studies consistently suggest that disruption of the intestinal microbial community is a common feature associated with disease progression and physiological stress. Shrimp diseases can be caused by viruses, bacteria, parasites, or complex interactions between pathogens, environmental stressors, and microbial imbalance. Major diseases affecting shrimp production include viral diseases such as white spot disease, bacterial diseases such as acute hepatopancreatic necrosis disease and vibriosis, parasitic infections caused by Enterocytozoon hepatopenaei, and multifactorial disorders such as white feces syndrome. A summary of major shrimp diseases, their causes, symptoms, and current control strategies is provided in S3 Table. However, most previous studies have focused primarily on changes in gut microbiota, whereas the interactions among oxidative stress, intestinal microbial dysbiosis, and environmental microbial communities remain poorly understood in shrimp affected by WFS.

Beyond host-associated factors, the aquaculture environment also plays a critical role in shaping disease dynamics. Water quality parameters, particularly those associated with nitrogen cycling, are closely linked to microbial processes and ecosystem stability in culture systems [3,21]. Sediments, as important reservoirs of microorganisms, participate in nutrient cycling and organic matter degradation and may act as sources of opportunistic pathogens [22,23]. Environmental microbial communities can influence the composition of intestinal microbiota through continuous host–environment interactions, yet their contribution to WFS has received relatively limited attention. In addition to host-associated microbiota, environmental microbial communities are increasingly recognized as important determinants of shrimp health. Sediment microorganisms regulate organic matter degradation, nitrogen cycling, and water quality, thereby influencing pathogen dynamics within aquaculture systems [21–23]. Recent studies have suggested that continuous microbial exchange occurs between shrimp and their surrounding environment, indicating that disease development should be considered within the context of host–environment interactions rather than host-associated microbiota alone.

Although previous studies have independently investigated pathogens, intestinal microbiota, oxidative stress, or environmental factors associated with WFS, few studies have simultaneously evaluated these components within the same production system. Previous studies have mainly focused on either intestinal microbiota alterations or pathogen-associated factors during WFS outbreaks. However, the simultaneous characterization of host oxidative status, intestinal microbial composition, sediment microbial communities, and water quality parameters within the same commercial culture system remains limited. Consequently, the interactions among host antioxidant status, intestinal microbial dysbiosis, sediment microbial communities, and water quality remain poorly understood. Addressing these knowledge gaps may improve our understanding of WFS pathogenesis and facilitate the development of integrated disease management strategies. The lack of longitudinal monitoring, mortality records, and comprehensive pathogen screening represents a limitation of the present study.

Therefore, the present study simultaneously investigated intestinal antioxidant capacity, intestinal microbiota, sediment microbial communities, and water quality characteristics in *L. vannamei* affected by WFS under commercial pond conditions. By integrating host physiological responses with environmental microbial dynamics, this study provides a comprehensive host–microbiota–environment perspective on WFS and identifies potential biomarkers for disease monitoring and aquaculture management. In addition, no histopathological examination was performed in the present study, which limits the pathological interpretation of WFS.

## 2. Materials and methods

### 2.1. Ethics statement

The animal protocol was approved by the Ethics Review Board of the Guangdong Ocean University. All procedures were performed according to the standards of the National Institutes of Health Guide for the Care and Use of Laboratory Animals (NIH Publication No. 8023, revised 1978) and relevant Chinese policies. All methods and procedures were performed according to relevant guidelines and regulations.

### 2.2. Sample collection

Recently, June to October has been identified as the period with a high incidence of WFS outbreaks in *L. vannamei* cultured in China. Shrimp samples were collected from four independent small-pond culture systems located at Haizhipeng Ecological Agriculture Co., Ltd., Zhanjiang City, Guangdong Province, China. Each pond was considered an independent biological replicate. The ponds had similar management conditions, including stocking density, feeding regime, aeration, and water exchange practices.

For each pond, shrimp exhibiting typical clinical signs of WFS were collected as the WFS group, while clinically healthy shrimp from the corresponding pond were collected as the control group. Three to five shrimp individuals were pooled to generate one biological sample per replicate. Each culture shed was stocked with 45,000 post-larvae. The shrimp were fed three times daily during the early culture stage and four times daily during the late stage. In intensive shrimp farming systems, WFS is more frequently observed during the middle-to-late culture period, particularly when shrimp biomass increases and environmental conditions become more variable. Aeration was supplied via air pumps, and 10% of the total water volume was exchanged every day. The sampled shrimp were in the rapid growth stage. Diagnosis was based on the combination of clinical signs, including white fecal strings, reduced feeding activity, empty intestine, and external observation. Because WFS is a multifactorial syndrome, molecular confirmation of a single pathogen was not used as the diagnostic criterion. Healthy shrimp without visible clinical signs were used as controls. Although EHP and Vibrio spp. have been reported to be associated with WFS outbreaks, WFS diagnosis in the present field investigation was based on typical clinical manifestations rather than pathogen-specific confirmation. Therefore, the collected shrimp were defined as WFS-associated shrimp rather than experimentally infected shrimp. On June 27, shrimp exhibiting typical clinical symptoms of WFS were collected; meanwhile, healthy shrimp were collected from the same pond during the same period as controls. The average body weight of the cultured shrimp used in this experiment was 16.20 ± 1.85 g. A total of eight independent biological replicates were set: four normal shrimp samples were designated as the control group (labeled CON1, CON2, CON3, and CON4), while diseased shrimp samples were labeled as the White Feces Syndrome group (labeled WFS1, WFS2, WFS3, and WFS4). WFS-associated ponds were identified based on farm history and typical clinical signs, including white fecal strings, reduced feeding activity, and abnormal swimming behavior. 3–5 healthy/diseased shrimp were mixed to form one sample per replicate. Pooling several shrimp individuals from the same pond was performed to reduce individual variation and obtain sufficient intestinal tissue for biochemical and microbiome analyses. The pond, rather than individual shrimp, was considered the experimental unit. Collected intestinal tissues were placed into cryotubes, rapidly frozen in liquid nitrogen, and then stored at −80°C for enzyme activity assays and microbiome studies. Sediment samples were collected from the surface layer (0–5 cm) at three randomly selected locations within each pond. Equal amounts of sediment from the subsampling sites were homogenized to generate one representative sediment sample per ponds. Approximately 5 g sediment was transferred into sterile tubes, immediately frozen in liquid nitrogen, and stored at −80°C until DNA extraction.

### 2.3. Water quality analysis

Water samples were collected from the same culture pond where shrimp samples were obtained. Four water samples were collected from different locations within the pond and treated as biological replicates for water quality analysis [24]. The determinations of nitrite, nitrate, total nitrogen, total phosphorus, and organic carbon were performed in accordance with China Marine Code Part 4: Seawater Analysis [25]. Nitrite, nitrate, total nitrogen, and total phosphorus were determined using an ultraviolet (UV) spectrophotometer, while organic carbon was measured with a total organic carbon (TOC) analyzer.

### 2.4. Antioxidant capacity assay

The intestinal samples stored in the refrigerator at 80 ℃ were thawed and weighed 0.5 g, then milled and diluted with phosphate buffer solution in the ratio of 1: 9 at 4 ℃, centrifuged at 3000g/min for 15 min at 4 ℃, and the supernatant was taken. The supernatant was collected for the determination of total antioxidant capacity (T-AOC, A015-2-1), superoxide dismutase (SOD, A001-3-2), glutathione peroxidase (GSH-Px, A005-1-1), and malondialdehyde (MDA, A003-1-2). The measurements were performed using assay kits (Nanjing Jiancheng Bioengineering Institute, Nanjing, China). All specific procedures and calculations were carried out in strict accordance with the instructions provided in the kits. The activities of SOD, GSH-Px, T-AOC, and MDA were determined using commercial assay kits according to the manufacturer's instructions and previously described methods [11,26–28].

### 2.5. DNA extraction and 16S rDNA Amplicon Sequencing

Total genome DNA from samples was extracted using the Mag-bind soil DNA kit (Omega), and the purity and concentration of DNA were tested. According to the selection of the sequencing region, the selected V3-V4 region (341F: CCTAYGGGRBGCASCAG, 806R: GGACTACNNGGGTATCTAAT) was amplified by PCR using specific primers with a barcode and high-fidelity DNA polymerase. PCR amplification was performed in a 25 μL reaction mixture containing 12.5 μL 2× PCR Master Mix, 0.2 μM of each primer and approximately 10 ng template DNA. The PCR program consisted of an initial denaturation at 98 °C for 30 s, followed by 30 cycles of denaturation at 98 °C for 10 s, annealing at 54 °C for 30 s, extension at 72 °C for 45 s, and a final extension at 72 °C for 5 min. PCR products were detected by 2% agarose gel electrophoresis, and the target fragments were cut and recovered by the Quant-iT PicoGreen dsDNA Assay Kit. Referring to the preliminary quantitative results of electrophoresis, the PCR amplification recovered products were detected and quantified with the Microplate reader (BioTek, FLx800) fluorescence quantitative system, and the corresponding proportions were mixed according to the sequencing requirements of each sample. The library was constructed using the TruSeq Nano DNA LT Library Prep Kit from Illumina. The constructed library is inspected by Agilent Bioanalyzer 2100 and Promega QuantiFluor. After the library is qualified, it is sequenced on an Illumina MiSeq platform using paired-end 2 × 250 bp reads.

### 2.6. Raw data processing and bioinformatics

Raw sequencing data were in FASTQ format. Paired-end reads were then preprocessed using Cutadapt software to detect and cut off the adapter. After trimming, paired-end reads were quality filtered (reads with Q score <20 were removed), denoised, merged and chimera sequences were removed using DADA2 in QIIME2 (2022.2). At last, the software outputs the representative reads and the ASV abundance table.

The representative read of each ASV was selected using the QIIME 2 package. All representative reads were annotated and blasted against the Silva database version 138 (16S rDNA) using classify-sklearn with the default parameters.

Calculate the α and β diversity indices with QIIME2 software. Alpha diversity indices (Shannon, Simpson, Chao1 and ACE) were calculated using QIIME2. Differences among groups were assessed using the Kruskal–Walli’s test followed by Dunn’s post hoc test. Statistical significance was set at p < 0.05. The richness and diversity of microbial communities in the sample can be reflected through the diversity analysis of a single sample (alpha diversity), including the use of a species accumulation box chart, a species diversity curve, and a series of statistical analysis indexes to evaluate the differences in species richness and diversity of microbial communities in each sample. Beta diversity differences were evaluated using Bray–Curtis distance matrices and tested by permutational multivariate analysis of variance (PERMANOVA) with 999 permutations. The sequencing data were processed using QIIME2 following established microbiome analysis protocols [29,30].

### 2.7. Functional prediction

PICRUSt2 (Phylogenetic Investigation of Communities by Recommendation of Unobserved States) is software that predicts functional abundance only based on the marker gene 16S sequence. It infers the gene function spectrum of their common ancestors based on the tree of OTUs in the Greengenes database and the gene information on OTUs, infers the gene function spectrum of other undetected species in the Greengenes database, builds the gene function prediction spectrum of the whole pedigree of the archaea and bacteria domain, and finally “maps” the composition of the flora obtained by sequencing into the database to predict the metabolic function of the flora. Functional profiles were predicted using PICRUSt2 based on ASV representative sequences and abundance tables generated by DADA2. The predicted metagenomes were annotated against the KEGG database. PICRUSt2 was performed according to the standard workflow, including sequence placement, hidden-state prediction, and pathway inference. OTUs were clustered using UPARSE [31]. Functional profiles were predicted using PICRUSt2 [32].

### 2.8. Statistical analysis

Statistical analyses were performed using SPSS 26.0 and R software. Data are presented as mean ± standard deviation (SD). Differences between the two groups were analyzed using independent sample t-tests when data met the assumptions of normality and homogeneity of variance; otherwise, the Mann–Whitney U test was applied. Alpha diversity indices were compared using the Mann–Whitney U test. Beta diversity was visualized using principal coordinate analysis (PCoA) based on Bray–Curtis distances, and differences in microbial community composition were evaluated using PERMANOVA with 999 permutations according to Anderson [33]. Spearman correlation analysis was performed to evaluate relationships among environmental factors, antioxidant parameters, and dominant microbial taxa. Correlation coefficients and *P* values were calculated, and false discovery rate (FDR) correction was applied for multiple comparisons. Differences were considered statistically significant at *P* < 0.05. Due to the observational nature and limited biological replicates, results were interpreted as associations rather than causal relationships.

## 3. Results

### 3.1 Clinical observations

Shrimp from WFS-associated ponds exhibited typical clinical signs, including white fecal strings floating on the water surface, reduced feeding activity, and sluggish swimming behavior compared with healthy shrimp. No obvious external shell lesions or body discoloration were observed in WFS-associated shrimp. In contrast, shrimp from healthy ponds showed normal feeding activity, active swimming behavior, and no visible white fecal strings.

### 3.2 Water quality parameters

As shown in Table 1 and S1 Fig. Compared with the CON group, the contents of nitrate and total nitrogen in the water samples of the WFS group were significantly decreased (nitrate: *P* = 0.030; total nitrogen: *P* = 0.006). However, organic carbon, nitrite, and total phosphorus in the water samples showed no significant differences.

**Table 1:** Comparison of water quality between the CON group and the WFS group (n = 4).

CON	15.20 ± 2.18	0.96 ± 0.12	16.37 ± 2.21	23.39 ± 1.39	4.69 ± 4.78
WFS	11.53 ± 1.25	3.36 ± 2.23	6.09 ± 1.63	12.89 ± 3.10	1.80 ± 0.45
*P*-value	0.065	0.136	0.03	0.006	0.356

### 3.3 Assessment of intestinal antioxidant capacity

As shown in Table 2 and S2 Fig, in contrast to healthy shrimp, compared with the CON group, WFS-affected shrimp showed significantly higher MDA levels, accompanied by significantly reduced activities of T-AOC, SOD, and GSH-Px.

**Table 2:** Comparison of antioxidant capacity between the CON group and the WFS group (n = 4).

CON	0.045 ± 0.009	1.330 ± 0.048	16.433 ± 0.349	2.905 ± 0.490
WFS	0.167 ± 0.013	1.124 ± 0.008	15.493 ± 0.281	2.056 ± 0.093
*P*-value	<0.001	0.002	0.022	0.042

### 3.4 Intestinal microbial analysis

As shown in Table 3 and Fig 1A; B; C; D, observed species, Chao 1, Shannon, and Simpson indices were the key metrics for alpha diversity analysis. The WFS group exhibited slightly higher observed species and Chao1 indices compared with the CON group; however, no significant differences were observed between groups (*P* > 0.05). Similarly, Shannon and Simpson indices also showed no significant differences (*P* > 0.05). Meanwhile, both the CON and WFS groups had Shannon index values > 3 and Simpson index values close to 1, which are indicative of high species richness and evenness, respectively, confirming the sufficient richness of the microbial communities.

**Table 3:** Alpha diversity measures of CON and WFS groups (n = 4) in intestinal.

Sample	Chao1	Shannon	Simpson
CON-1	2232.8919	8.6154	0.99
CON-2	1954.2857	8.1411	0.9836
CON-3	1892.4388	8.0971	0.9858
CON-4	2323.75	8.6647	0.9888
WFS-1	2253.5508	8.4898	0.9899
WFS-2	2418.1515	8.7974	0.9926
WFS-3	2297.0079	8.3595	0.9865
WFS-4	2658.5294	8.8406	0.9918

**Fig 1:**
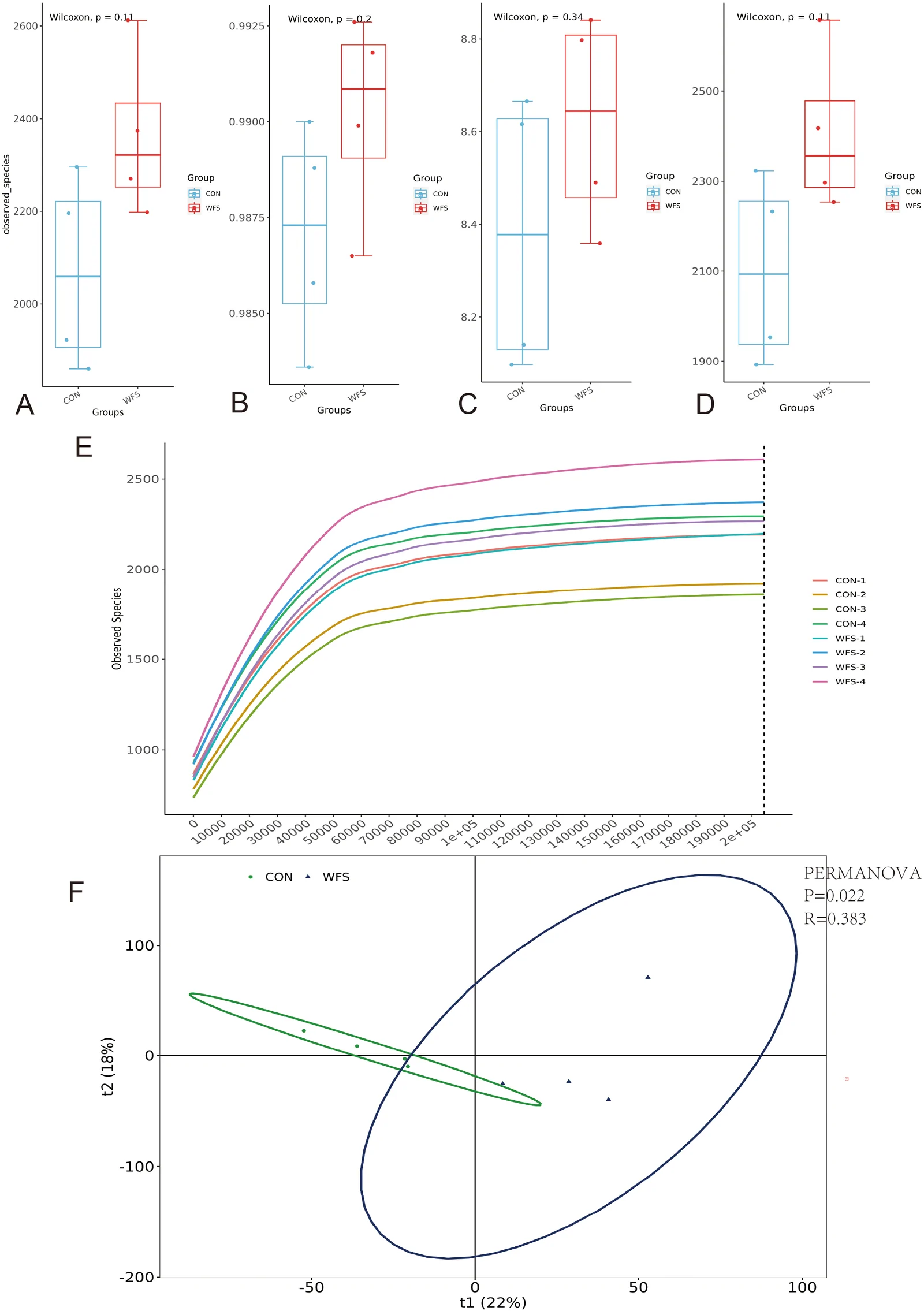
Intestinal Alpha and Beta diversity analysis (n = 4). (A) Observed species, (B) Shannon, (C) Simpson and (D) Chao1; (E) Rarefaction curve; (F) Principal component analysis. Note: CON, control group; WFS, white feces group.

Furthermore, the rarefaction curves of 8 samples plateaued (Fig 1E), indicating that the sequencing depth was sufficient to capture the full diversity of the microbial communities in these samples. Furthermore, PCA analysis (Fig 1F) showed that the first two principal components (PC1 and PC2) explained 22% and 18% of the total variation, respectively. The WFS and CON groups exhibited clear separation in microbial community composition. PERMANOVA further confirmed that the overall microbial community structure differed significantly between groups (R^2^ = 0.407, P = 0.022).

At the phylum level (Fig 2A), the relative abundances of Firmicutes, Patescibacteria, and Bacteroidota were significantly increased in WFS-associated samples, while those of Actinobacteriota, Chloroflexi, and Verrucomicrobiota were significantly decreased. Additionally, three phyla, namely Proteobacteria, Actinobacteriota, and Firmicutes, accounted for more than 1% of the total sequences and were therefore considered core and dominant phyla. At the family level (Fig 2B), the relative abundance of Intrasporangiaceae was significantly decreased in the WFS-infected group, while that of Clostridiaceae was significantly increased. At the genus level (Fig 3A), compared with the CON group, the relative abundances of AKYG1722 and Tessaracoccus were significantly decreased in the WFS-infected group. According to the Venn diagram (Fig 3B), 2120 species were shared between the CON and WFS groups. The WFS group contained more unique microbial features than the CON group (2607 vs. 1999), although this difference was descriptive rather than statistically tested.

**Fig 2:**
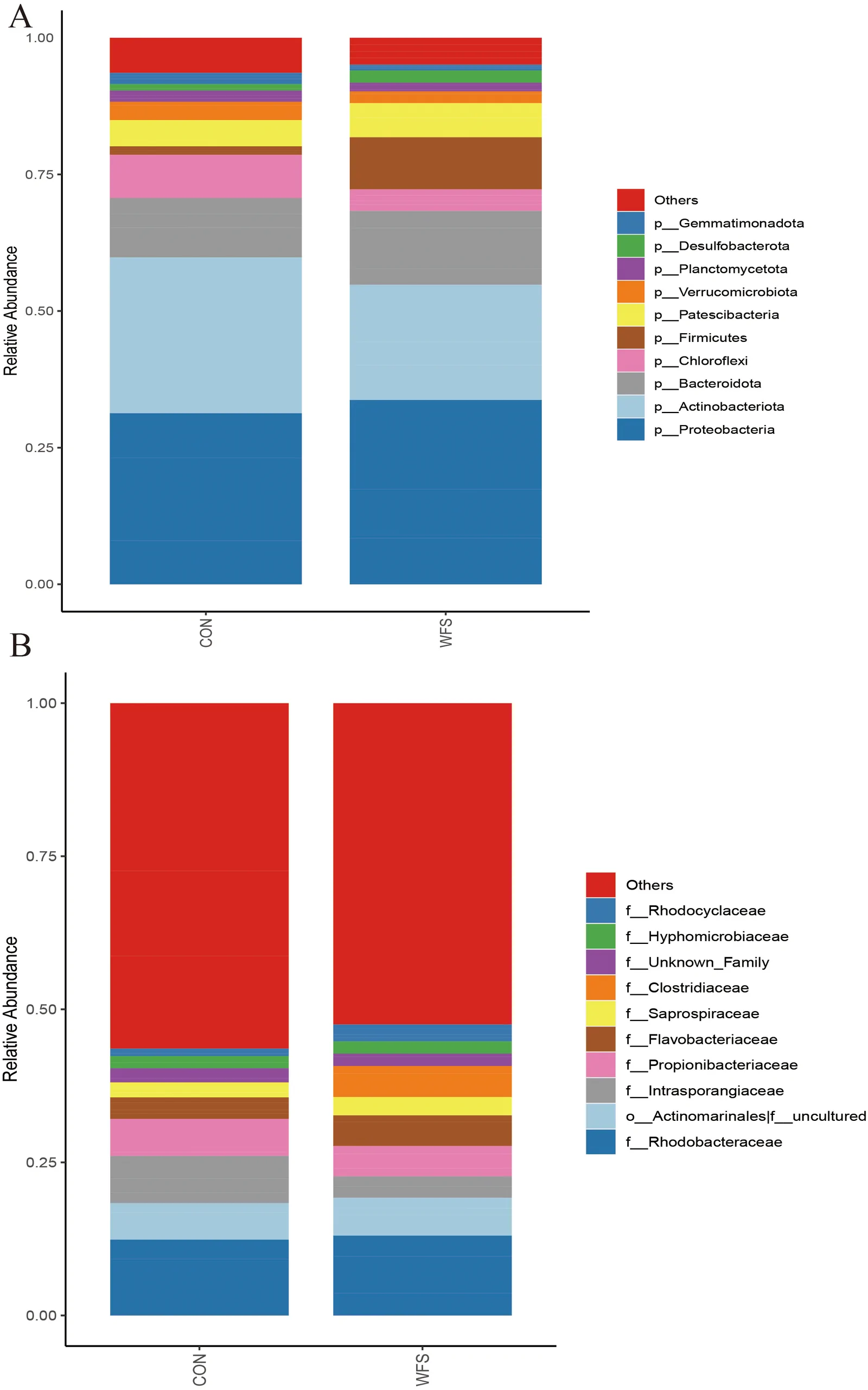
Taxonomy of relative abundance of intestinal microbiota at (A) Phylum, (B) Family. Note: CON, control group; WFS, white feces group. “Others” represents the “sum of species with relative abundance < 1%”.

**Fig 3:**
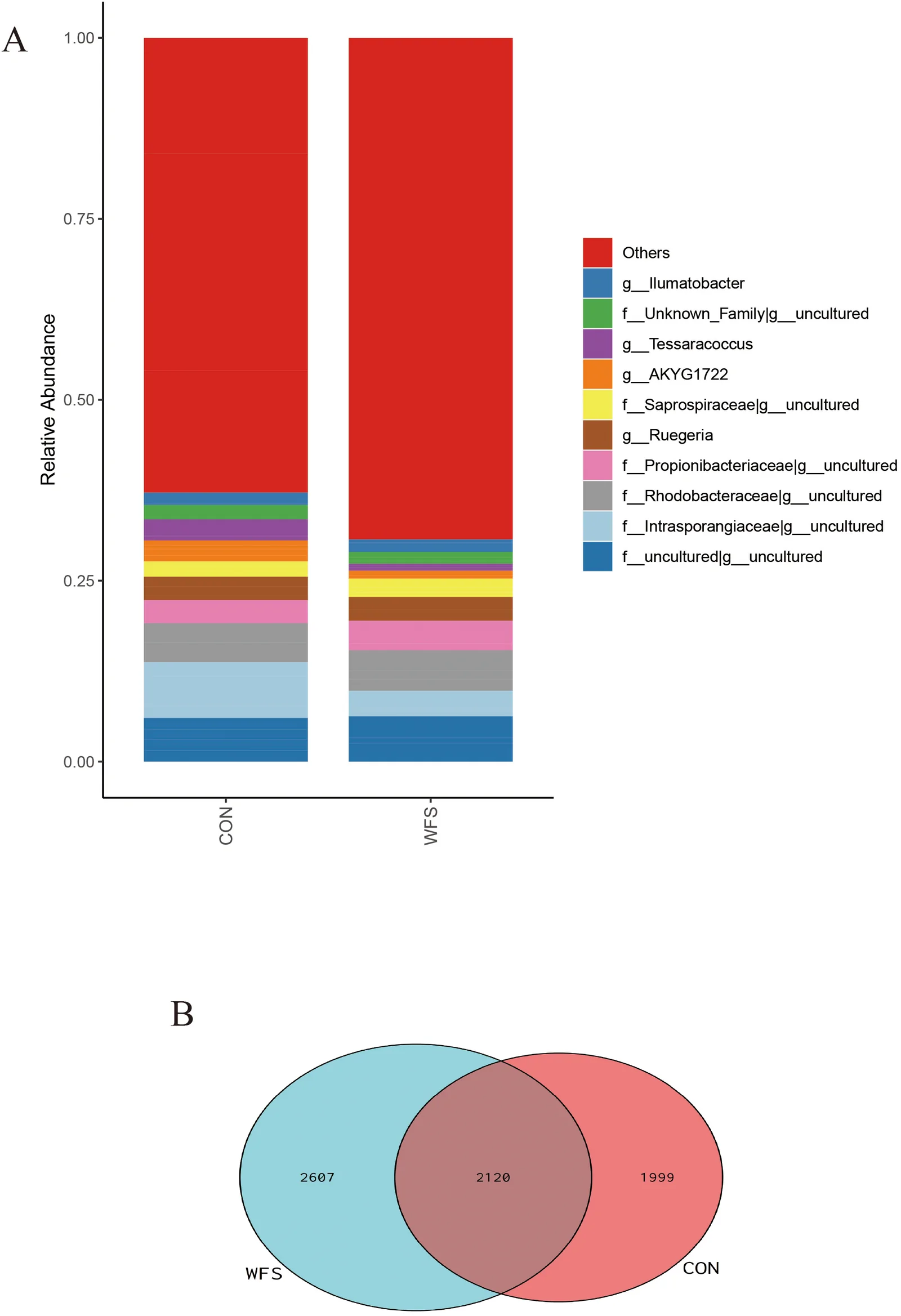
Taxonomy of relative abundance of intestinal microbiota at (A) genus level and (B) Venn diagram. Note: CON, control group; WFS, white feces group. “Others” represents the “sum of species with relative abundance < 1%”.

The alterations of the intestinal microbiota functions in the CON and WFS groups were examined using PICRUSt. As shown in Fig 4A, the top five predicted functions mainly were amino acid metabolism, carbohydrate metabolism, translation, membrane transport, and metabolism of cofactors and vitamins at level 2. Amino acid metabolism and carbohydrate metabolism were significantly down-regulated (Fig 4B).

**Fig 4:**
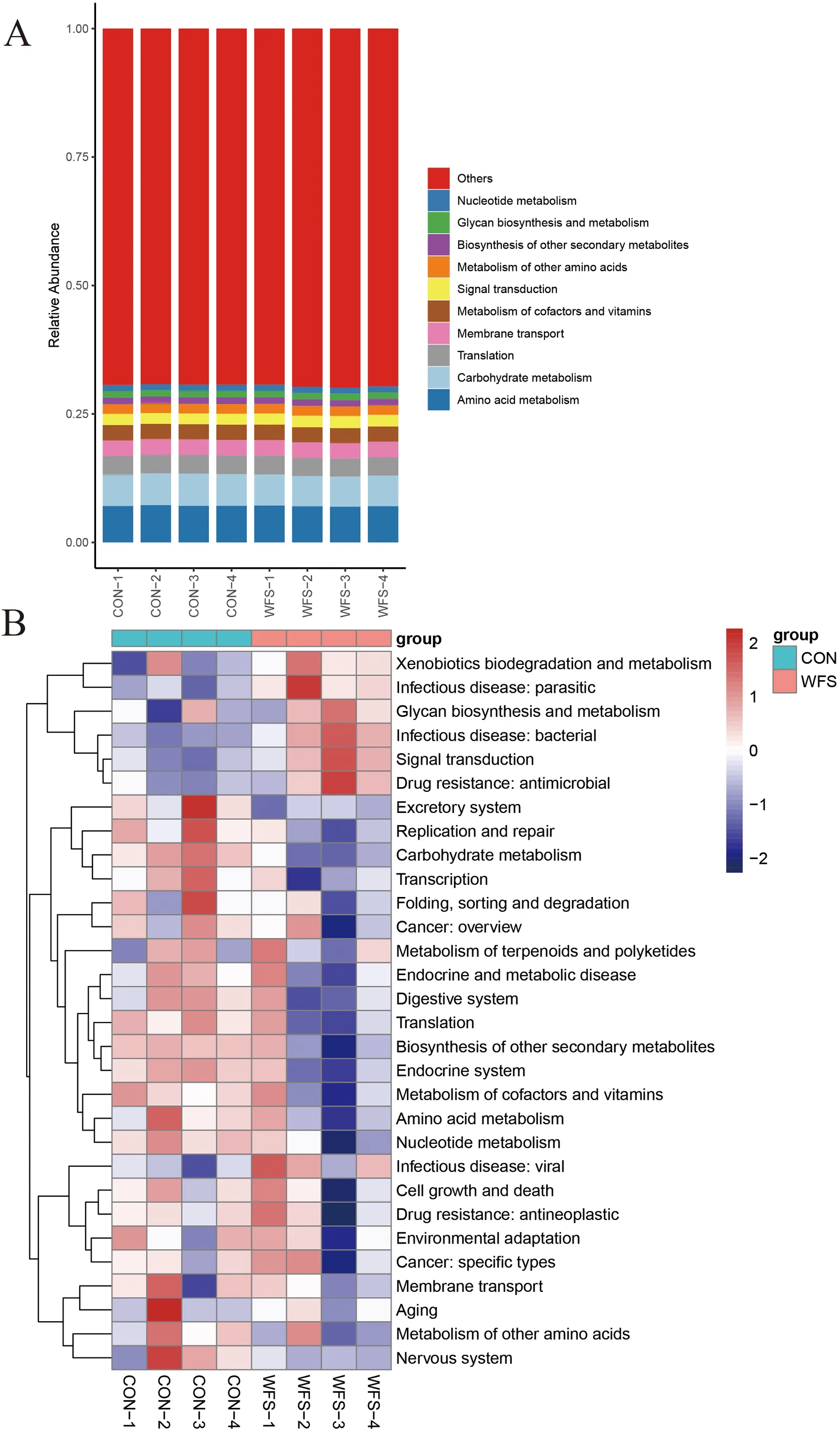
Predicted function at level 2 in KEGG metabolism pathway: (A) Relative abundance of the top 10, (B) Level 2 KEGG functional prediction relative abundance heatmap. Note: CON, control group; WFS, white feces group. “Others” represents the “sum of species with relative abundance < 1%”.

### 3.5 Sediment microbial diversity analysis

As presented in Table 4 and Fig 5A; B; C; D, relative to the CON group, the WFS group showed elevated observed species and Chao 1 values, reflecting a higher level of bacterial community richness. Notably, the Shannon index exceeded 3 and the Simpson index approximated 1 in both groups, which are typical indicators of high species richness and evenness, respectively, validating the sufficient richness of the microbial communities.

**Table 4:** Alpha diversity measures of CON and WFS groups (n = 4) in sediment.

Sample	Chao1	Shannon	Simpson
CON-1	1702.2192	7.6431	0.9733
CON-2	1723.6154	8.0233	0.9895
CON-3	1714.0196	8.0529	0.989
CON-4	1912.6026	8.3709	0.9914
WFS-1	2296.9718	7.3599	0.9652
WFS-2	2722.4437	9.7969	0.9967
WFS-3	5664.1833	10.2668	0.9964
WFS-4	4686.2377	9.1204	0.986

**Fig 5:**
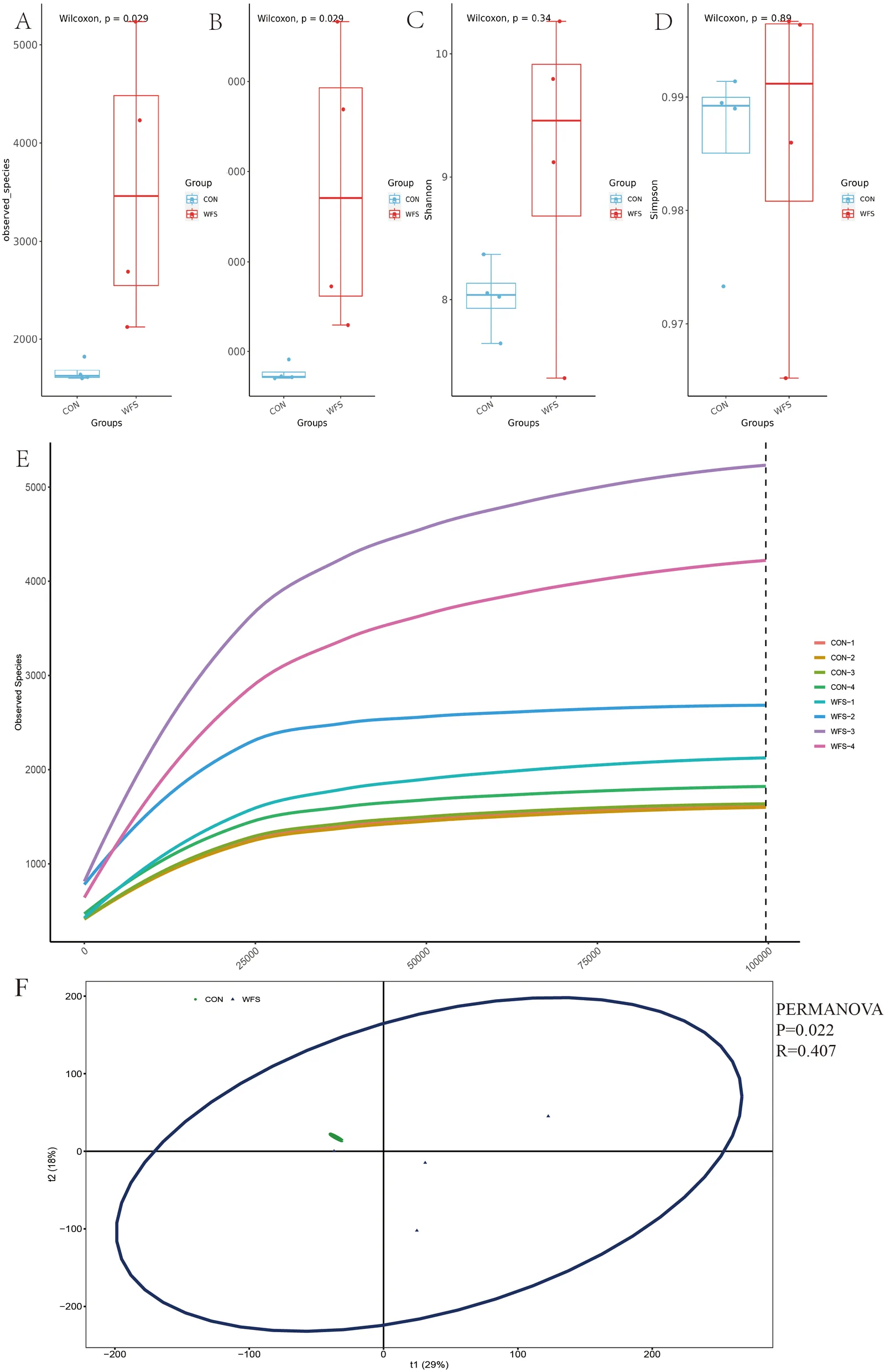
Sediment alpha and beta diversity analysis (n = 4). (A) Observed species, (B) Chao1, (C) Shannon, (D) Simpson, (E) Rarefaction curve, and (F) Principal component analysis. Note: CON, control group; WFS, white feces group.

Additionally, the rarefaction curves of all eight samples leveled off (Fig 5E), indicating that the sequencing depth was sufficient to capture the full diversity of the microbial communities in these samples. Furthermore, in the PCA (Fig 5F), t1 and t2 accounted for 29% and 18% of the total variation in beta diversity, respectively, which demonstrated that the sediment samples from WFS ponds exhibited a higher degree of dispersion than the healthy samples.

At the phylum level (Fig 6A), the relative abundance of Firmicutes, Chloroflexota, and Acidobacteriota in sediment samples from WFS ponds was significantly increased, while that of Proteobacteria, Actinobacteriota, and Bacteroidota was significantly decreased. At the family level (Fig 6B), the relative abundance of Lzemplasmataceae, Bacillaceae, JG30-KF-AS9, and Proteinivoracales in sediment samples from WFS ponds increased significantly, while that of Rhodobacteraceae, Actinomarinales, and Sulfurimonadaceae decreased significantly. At the genus level (Fig 7A), the relative abundance of JG30-KF-AS9, Lzemaplasma, and Acidothermus in sediment samples from WFS ponds increased significantly, while that of Sulfurimonas, Rhodobacteraceae, Desulfotignum, and Ruegeria decreased significantly. According to the Venn diagram (Fig 7B), it was shown that the 1436 species were present in both the CON and WFS groups. The WFS group contained more unique microbial features than the CON group (7238 vs. 2466), although this difference was descriptive rather than statistically tested. And JG30-KF-AS9 was identified as a dominant bacterial genus-level taxon in WFS-affected shrimp.

**Fig 6:**
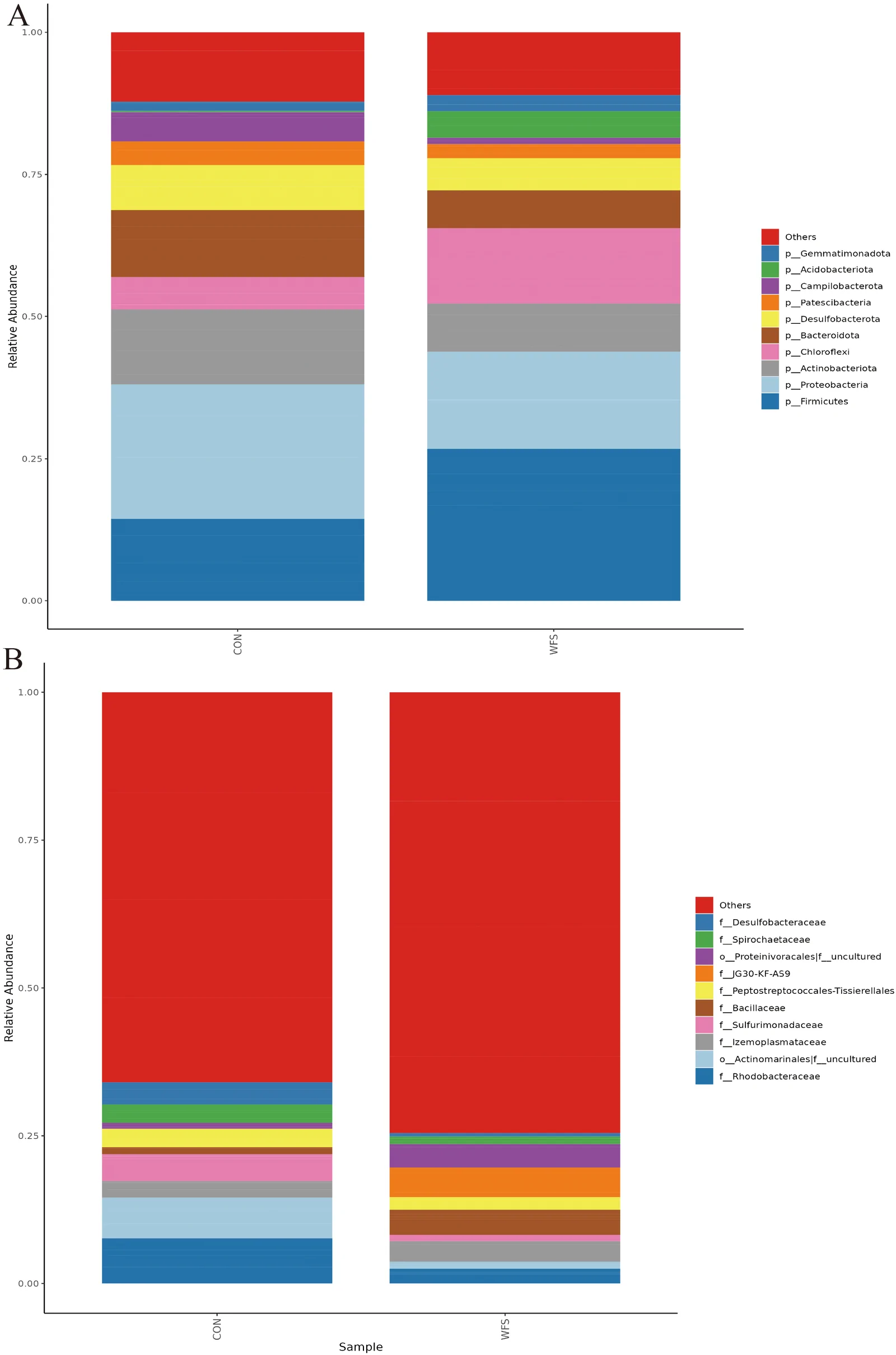
Taxonomy of relative abundance of sediment microbiota at (A) Phylum and (B) Family. Note: CON, control group; WFS, white feces group. “Others” represents the “sum of species with relative abundance < 1%”.

**Fig 7:**
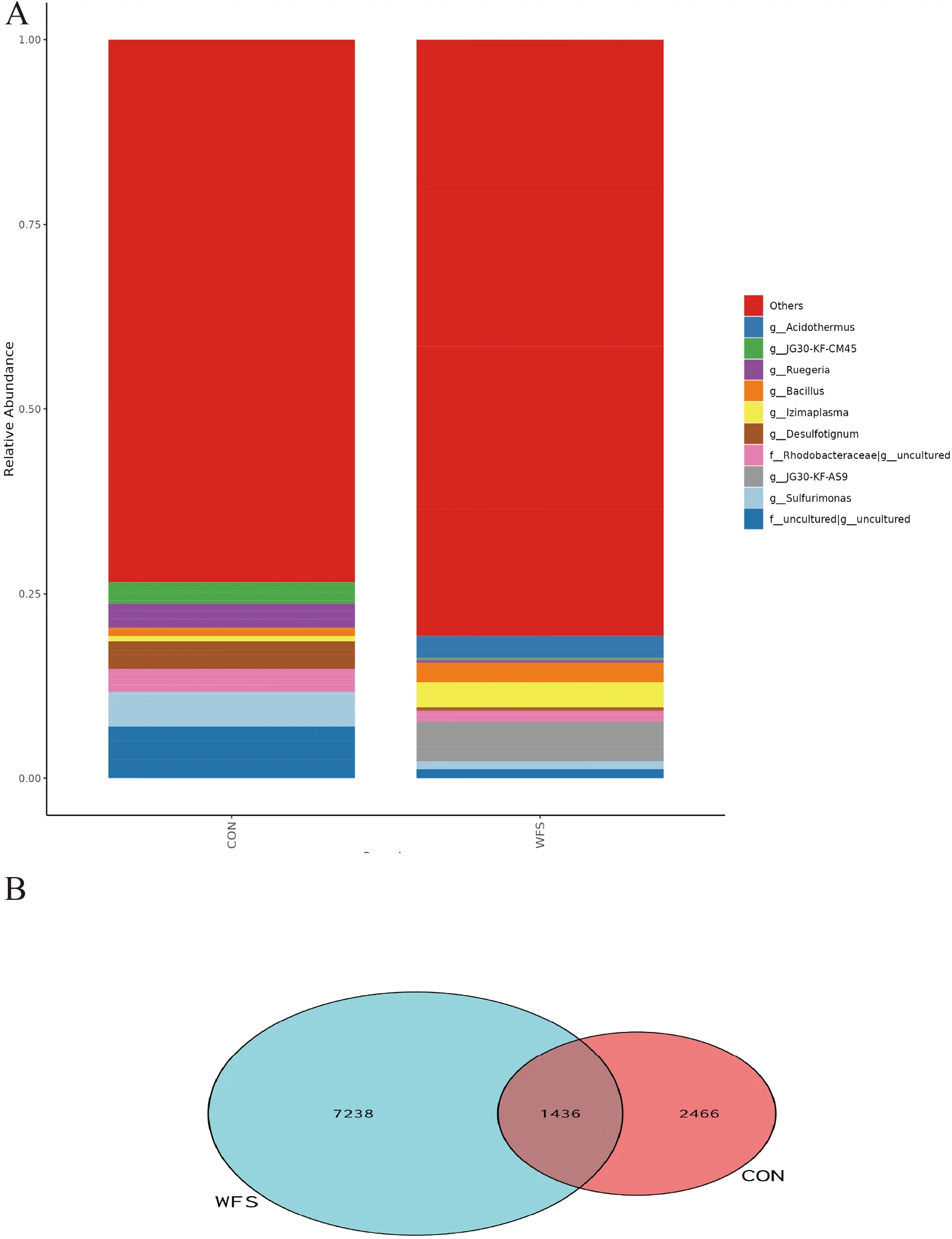
Taxonomy of relative abundance of sediment microbiota at (A) genus levels and (B) Venn diagram. Note: CON, control group; WFS, white feces group. “Others” represents the “sum of species with relative abundance < 1%”.

### 3.6 Sequence quality

After trimming the low-quality sequences, primers, and adapters, a total of 2,148,783 and 2,315,483 high-quality reads were generated using DADA2 with the default parameters in QIIME2, respectively. These reads served as representative reads for the determination of ASVs in shrimp intestinal samples and shrimp aquaculture sediment samples. Basic sequence statistics are presented in Tables 5 and 6. It was observed that in both experiments, the Q2 values of the CON and WFS groups exceeded 98%, and the Q3 values were greater than 95%, which indicated that the reads obtained in these two experiments were of high quality and credibility.

**Table 5:** Sequence statistics of CON and WFS groups in intestinal.

Name	Clean_reads	Max_len	Min_len	Avg_len	Q20(%)	Q30(%)
CON-1	289339	243	202	223.4	98.82	96.97
CON-2	253763	243	222	223.4	98.78	96.85
CON-3	258874	243	134	223.7	98.87	97.04
CON-4	233938	243	185	223.5	98.94	97.23
WFS-1	296295	243	222	223.3	98.65	96.38
WFS-2	269235	243	51	223.4	98.8	96.88
WFS-3	272000	243	222	223.4	98.81	96.92
WFS-4	275339	243	134	223.4	98.83	97

**Table 6:** Sequence statistics of CON and WFS groups in sediment.

Name	Clean_reads	Max_len	Min_len	Avg_len	Q20(%)	Q30(%)
CON-1	237168	243	192	223.3	98.6	96.27
CON-2	276529	243	207	223.3	98.38	95.67
CON-3	196446	243	67	223.3	98.48	95.95
CON-4	234674	243	206	223.3	98.52	96.07
WFS-1	242246	243	222	223.3	98.57	96.19
WFS-2	128620	243	222	223.3	98.39	95.71
WFS-3	491589	243	102	223.3	98.41	95.76
WFS-4	508211	243	67	223.3	98.42	95.79

## 4. Discussion

In the present study, WFS was associated with oxidative stress, intestinal microbial dysbiosis, and alterations in sediment microbial communities. In aquaculture systems, nitrogen dynamics are closely associated with feeding activity, metabolic waste production, and microbial transformation processes [3,21]. In the present study, nitrate and total nitrogen concentrations were significantly lower in the WFS group compared with the control group, whereas other parameters showed no marked differences. This pattern may reflect reduced feed intake and impaired nutrient assimilation in diseased shrimp, leading to decreased excretion of nitrogenous wastes. Consequently, the availability of substrates for nitrification may be limited, resulting in reduced nitrate accumulation in the culture water. These findings indicate that host physiological impairment during WFS may indirectly influence nitrogen cycling within the aquaculture environment.

The occurrence of WFS is unlikely to be attributable to a single factor but may result from the interaction among environmental stress, microbial imbalance, and host physiological dysfunction. In intensive shrimp farming systems, environmental deterioration and microbial community shifts may disturb intestinal microbial homeostasis, which could subsequently impair nutrient metabolism, antioxidant defense, and immune regulation. Conversely, physiological impairment of shrimp may further modify the surrounding microbial environment through altered feeding behavior, excretion patterns, and organic matter cycling. Therefore, WFS should be considered as a complex host–microbiota–environment interaction rather than a disease caused by a single pathogenic factor.

The intestinal antioxidant system plays a central role in maintaining cellular homeostasis under stress conditions. In this study, WFS-affected shrimp exhibited elevated MDA levels together with reduced activities of T-AOC, SOD, and GSH-Px, suggesting a disruption of redox balance. This pattern indicates that oxidative stress may be involved in the pathological process of WFS. One possible explanation is that pathogenic challenge or environmental stress induces excessive production of reactive oxygen species (ROS), which can overwhelm antioxidant defenses and result in lipid peroxidation [11,34]. Alternatively, decreased enzyme activities may reflect suppression of antioxidant systems at the molecular level. Similar oxidative disturbances have been widely reported in aquatic animals under disease or stress conditions, supporting the role of redox imbalance in host physiological dysfunction.

The intestinal microbiota is a key determinant of host health and is highly sensitive to environmental and physiological perturbations [12,35]. Although no significant differences were observed in alpha diversity indices between groups, clear shifts in microbial community composition were detected in WFS shrimp. This suggests that WFS is characterized more by structural reorganization than by a simple loss of diversity. The increased richness observed in the WFS group may indicate colonization by opportunistic or environmental microorganisms, potentially facilitated by weakened host defenses or disruption of the original microbial equilibrium [17,36].

At the phylum level, the increased abundance of Firmicutes and Bacteroidota, along with the reduction of Actinobacteriota and other taxa, indicates a shift in microbial functional potential. Firmicutes include both beneficial fermentative bacteria and opportunistic pathogens, and their enrichment may reflect altered intestinal conditions under stress [37]. In contrast, Actinobacteriota are often associated with antimicrobial activity and maintenance of microbial homeostasis, and their decline may be associated with altered microbial homeostasis [38]. These compositional changes suggest a transition from a relatively stable microbial community toward a more disturbed and potentially dysbiotic state.

At finer taxonomic resolution, several bacterial taxa exhibited altered abundances between the CON and WFS groups. Specifically, Clostridiaceae was significantly enriched in WFS shrimp, increasing from 0.04% in the CON group to 5.06% in the WFS group (Wilcoxon rank-sum test, *p* = 0.029). In contrast, Intrasporangiaceae showed a significant reduction, decreasing from 7.63% in the CON group to 3.49% in the WFS group (*p* = 0.029). Tessaracoccus also showed a decreasing trend (from 2.85% in CON to 0.93% in WFS), although this difference was not statistically significant (*p* = 0.200). These results indicate that WFS-associated intestinal dysbiosis involves shifts in specific bacterial groups rather than uniform changes across all taxa. Some members of Clostridiaceae are known to produce metabolites that can damage intestinal tissues or trigger inflammatory responses [39]. In contrast, taxa that declined in the WFS group may contribute to microbial balance or pathogen suppression [40]. The expansion of unique microbial taxa observed in WFS shrimp also suggests increased exposure to environmental microorganisms, which may further destabilize the intestinal ecosystem and exacerbate disease progression.

Functional prediction analysis revealed that pathways related to amino acid and carbohydrate metabolism were significantly downregulated in the WFS group. This reduction in metabolic potential may reflect impaired digestive and absorptive capacity, which is consistent with the reduced growth performance typically observed in diseased shrimp [17,41]. These findings suggest that WFS not only alters microbial composition but also disrupts the functional capabilities of the intestinal microbiome, potentially affecting host nutrient utilization and resilience to stress. These predicted functional alterations may indicate reduced metabolic capacity of the intestinal microbial community; however, PICRUSt2 predictions represent potential functional changes rather than direct measurements of microbial activity. Therefore, metagenomic or metabolomic analyses are required to validate these predicted metabolic alterations.

In addition to intestinal changes, the sediment microbial community also exhibited substantial alterations under WFS conditions. Sediments serve as important reservoirs of microorganisms and play key roles in nutrient cycling and organic matter degradation in aquaculture systems [22,23]. The increased richness and compositional shifts observed in the WFS group may be associated with changes in organic matter input, particularly from uneaten feed and host excreta. These changes can reshape microbial community structure and potentially influence water quality and microbial exposure to shrimp.

The observed decrease in Proteobacteria and other functionally important taxa in sediments may have implications for nutrient cycling processes, particularly those related to nitrogen transformation [42]. environmental microbial disturbances may be associated with differences in intestinal microbiota composition. In addition, the decline of sulfur-cycling microorganisms, such as Sulfurimonas, may lead to the accumulation of toxic compounds [43], thereby increasing environmental stress on cultured shrimp.

Collectively, these findings support a potential interaction model in which environmental microbial disturbances and altered water conditions may influence intestinal microbial stability, while intestinal dysbiosis and oxidative imbalance may weaken shrimp physiological resilience. Nevertheless, further experimental validation is required to determine the directionality and causal relationships among these factors.

Overall, the results of this study highlight the interconnected relationships among host physiological status, intestinal microbiota, and environmental microbial communities during WFS outbreaks. Rather than being driven by a single factor, WFS appears to involve complex interactions among oxidative stress, microbial dysbiosis, and environmental changes. This integrated perspective provides a more comprehensive understanding of disease mechanisms and underscores the importance of coordinated management of host health and environmental conditions in intensive aquaculture systems.

Despite these findings, several limitations should be acknowledged. Although four independent ponds were included in this study, the relatively limited number of pond replicates and the observational nature of the field investigation may restrict the generalization of the findings. Further studies involving larger numbers of farms and controlled experiments are required. In addition, the cross-sectional design does not allow causal relationships to be established. Furthermore, functional predictions based on 16S rRNA data provide only indirect evidence of microbial activity and require validation through metagenomic or transcriptomic approaches. Future studies incorporating controlled experiments and multi-omics analyses would help to clarify the mechanisms underlying WFS and strengthen the conclusions drawn here. Although histopathological examination was not included, the observed oxidative imbalance and microbial dysbiosis provide physiological evidence associated with WFS progression. Future studies incorporating histological evaluation are needed. In addition, although EHP and Vibrio spp. have been reported as potential contributors to WFS development, the present study did not perform molecular detection of EHP infection or quantitative analysis of Vibrio populations. Therefore, the contribution of these potential pathogens to the observed microbial dysbiosis and oxidative stress responses remains unclear. Future studies incorporating pathogen detection and controlled infection experiments are required to further elucidate the etiology of WFS.

## 5. Conclusion

This study demonstrated that WFS is associated with oxidative stress, intestinal microbial dysbiosis, and alterations in environmental microbial communities. These findings suggest that host physiological status and environmental microbial dynamics are closely interconnected during WFS outbreaks. Future studies combining controlled infection experiments and multi-omics approaches are required to verify the underlying mechanisms.

## Supporting information

S1 Fig Comparison of water quality between the CON group and the WFS group. (DOCX)

S2 Fig Comparison of antioxidant capacity between the CON group and the WFS group. (DOCX)

S1 Table List of abbreviations used in this manuscript. (DOCX)

S2 Table Differences between intestinal and environmental microbial communities and their potential relationships with WFS development and shrimp performance. (DOCX)

S3 Table Major diseases affecting shrimp aquaculture, their causes, symptoms, and current control strategies. (DOCX)

## References

[1] FAO. The state of world fisheries and aquaculture 2023: Towards sustainable and equitable blue transformations. Rome: Food and Agriculture Organization (FAO); 2023.

[2] Roy LA, Davis DA, Saoud IP, Boyd CA, Pine HJ, Boyd CE. Shrimp culture in inland low salinity waters. Rev Aquac. 2010;2(4):191–208. doi: 10.1111/j.1753-5131.2010.01036.x

[3] Dauda AB, Ajadi A, Tola-Fabunmi AS, Akinwole AO. Waste production in aquaculture: Sources, components and managements in different culture systems. Aquac Fish. 2019;4(3):81–8. doi: 10.1016/j.aaf.2018.10.002

[4] Palaniappan S, Bagthasingh C, Panchavarnam S, Mulloorpeedikayil RG, Loganathan S, Thirumal I, et al. Etiological factors driving white feces syndrome in farmed Pacific whiteleg shrimp, Penaeus vannamei in Tamil Nadu, India. Aquacult Int. 2024;32:4773–97. doi: 10.1007/s10499-024-01401-x

[5] Piamsomboon P, Han JE. White feces syndrome, a multifactorial syndrome of cultured shrimp: A mini review. Fishes. 2022;7(6):339. doi: 10.3390/fishes7060339

[6] Priya PS, Vaishnavi S, Sreekutty AR, Sudhakaran G, Arshad A, Arockiaraj J. White feces syndrome in shrimp: Comprehensive understanding of immune system responses. Fish Shellfish Immunol. 2024;151:109704. doi: 10.1016/j.fsi.2024.109704 38880362

[7] Tangprasittipap A, Srisala J, Chouwdee S, Somboon M, Chuchird N, Limsuwan C, et al. The microsporidian Enterocytozoon hepatopenaei is not the cause of white feces syndrome in whiteleg shrimp Penaeus (Litopenaeus) vannamei. BMC Vet Res. 2013;9:139. doi: 10.1186/1746-6148-9-139 23856195 PMC3717009

[8] Prachumwat A, Munkongwongsiri N, Eamsaard W, Lertsiri K, Flegel TW, Stentiford GD. A potential prokaryotic and microsporidian pathobiome that may cause shrimp white feces syndrome (WFS). bioRxiv. 2021:2021.05.23.445355. doi: 10.1101/2021.05.23.445355

[9] Munkongwongsiri N, Prachumwat A, Eamsaard W, Lertsiri K, Flegel TW, Stentiford GD, et al. *Propionigenium* and *Vibrio* species identified as possible component causes of shrimp white feces syndrome (WFS) associated with the microsporidian *Enterocytozoon hepatopenaei*. J Invertebr Pathol. 2022;192:107784. doi: 10.1016/j.jip.2022.107784 35659607

[10] Birben E, Sahiner UM, Sackesen C, Erzurum S, Kalayci O. Oxidative stress and antioxidant defense. World Allergy Organ J. 2012;5:9–19. doi: 10.1097/WOX.0b013e3182439613 PMC3488923 23268465

[11] Lushchak VI. Free radicals, reactive oxygen species, oxidative stress and its classification. Chem Biol Interact. 2014;224:164–75. doi: 10.1016/j.cbi.2014.10.016 25452175

[12] Ghanbari M, Kneifel W, Domig KJ. A new view of the fish gut microbiome: Advances from next-generation sequencing. Aquaculture. 2015;448:464–75. doi: 10.1016/j.aquaculture.2015.06.033

[13] Butt RL, Volkoff H. Gut microbiota and energy homeostasis in fish. Front Endocrinol. 2019. doi: 10.3389/fendo.2019.00009 PMC6353785 30733706

[14] Rooks MG, Garrett WS. Gut microbiota, metabolites and host immunity. Nat Rev Immunol. 2016;16(6):341–52. doi: 10.1038/nri.2016.42 27231050 PMC5541232

[15] Sommer F, Bäckhed F. The gut microbiota — masters of host development and physiology. Nat Rev Microbiol. 2013;11(4):227–38. doi: 10.1038/nrmicro2974 23435359

[16] Holt CC, Bass D, Stentiford GD, van der Giezen M. Understanding the role of the shrimp gut microbiome in health and disease. J Invertebr Pathol. 2021;186:107387. doi: 10.1016/j.jip.2020.107387 32330478

[17] Dai W-F, Zhang J-J, Qiu Q-F, Chen J, Yang W, Ni S, et al. Starvation stress affects the interplay among shrimp gut microbiota, digestion and immune activities. Fish Shellfish Immunol. 2018;80:191–9. doi: 10.1016/j.fsi.2018.05.040 29803665

[18] Zhou L, Chen C, Xie J, Xu C, Zhao Q, Qin JG, et al. Intestinal bacterial signatures of the “cotton shrimp-like” disease explain the change of growth performance and immune responses in Pacific white shrimp (*Litopenaeus vannamei*). Fish Shellfish Immunol. 2019;92:629–36. doi: 10.1016/j.fsi.2019.06.054 31265910

[19] Xv K, Zhang S, Pang A, Wang T, Dong S, Xv Z, et al. White feces syndrome is closely related with hypoimmunity and dysbiosis in *Litopenaeus vannamei*. Aquac Rep. 2024;38:102329. doi: 10.1016/j.aqrep.2024.102329

[20] Wang T, Pang A, Xv K, Zhang X, Luthfiah A, Jiang Y, et al. Analysis of intestinal metabolites in *Litopenaeus vannamei* infected with white faeces syndrome based on untargeted metabolomics. Comp Biochem Physiol Part D Genomics Proteomics. 2025;55:101462. doi: 10.1016/j.cbd.2025.101462 40120225

[21] Ebeling JM, Timmons MB, Bisogni JJ. Engineering analysis of the stoichiometry of photoautotrophic, autotrophic, and heterotrophic removal of ammonia–nitrogen in aquaculture systems. Aquaculture. 2006;257(1–4):346–58. doi: 10.1016/j.aquaculture.2006.03.019

[22] Ape F, Manini E, Quero GM, Luna GM, Sarà G, Vecchio P, et al. Biostimulation of in situ microbial degradation processes in organically-enriched sediments mitigates the impact of aquaculture. Chemosphere. 2019;226:715–25. doi: 10.1016/j.chemosphere.2019.03.178 30959456

[23] Song C, Zhong L, Liu Y, Yin Y, Zhang C, Zhang X, et al. Spatial and temporal succession of bacterial communities in three artificial fishponds. Aquac Res. 2019;50(10):2793–801. doi: 10.1111/are.14231

[24] Badan Standarisasi Nasional. Tentang Kualitas Air Laut (SNI 6964.8:2015); 2015.

[25] CN-GB. GB17378.4; 2007.

[26] Fridovich I. Superoxide radical and superoxide dismutases. In: Gilbert DL, editor. Oxygen and living processes. New York, NY: Springer; 1981. pp. 250–72. doi: 10.1007/978-1-4612-5890-2_13

[27] Esterbauer H, Schaur RJ, Zollner H. Chemistry and biochemistry of 4-hydroxynonenal, malonaldehyde and related aldehydes. Free Radic Biol Med. 1991;11(1):81–128. doi: 10.1016/0891-5849(91)90192-6 1937131

[28] Prior RL, Cao G. In vivo total antioxidant capacity: Comparison of different analytical methods. Free Radic Biol Med. 1999;27(11–12):1173–81. doi: 10.1016/s0891-5849(99)00203-8 10641708

[29] Caporaso JG, Kuczynski J, Stombaugh J, Bittinger K, Bushman FD, Costello EK, et al. QIIME allows analysis of high-throughput community sequencing data. Nat Methods. 2010;7(5):335–6. doi: 10.1038/nmeth.f.303 20383131 PMC3156573

[30] Bolyen E, Rideout JR, Dillon MR, Bokulich NA, Abnet CC, Al-Ghalith GA, et al. Reproducible, interactive, scalable and extensible microbiome data science using QIIME 2. Nat Biotechnol. 2019;37(8):852–7. doi: 10.1038/s41587-019-0209-9 31341288 PMC7015180

[31] Edgar RC. UPARSE: Highly accurate OTU sequences from microbial amplicon reads. Nat Methods. 2013;10(10):996–8. doi: 10.1038/nmeth.2604 23955772

[32] Douglas GM, Maffei VJ, Zaneveld JR, Yurgel SN, Brown JR, Taylor CM, et al. PICRUSt2 for prediction of metagenome functions. Nat Biotechnol. 2020;38(6):685–8. doi: 10.1038/s41587-020-0548-6 32483366 PMC7365738

[33] Anderson MJ. A new method for non‐parametric multivariate analysis of variance. Aust N Z J Stat. 2026 [cited 28 July 2026]. Available from: https://onlinelibrary.wiley.com/doi/10.1111/j.1442-9993.2001.01070.pp.x

[34] Martínez-Álvarez RM, Morales AE, Sanz A. Antioxidant defenses in fish: Biotic and abiotic factors. Rev Fish Biol Fisheries. 2005;15:75–88. doi: 10.1007/s11160-005-7846-4

[35] Guinane CM, Cotter PD. Role of the gut microbiota in health and chronic gastrointestinal disease: Understanding a hidden metabolic organ. SAGE J. 2013. doi: 10.1177/1756283X13482996 PMC3667473 23814609

[36] Xiong J, Zhu J, Dai W, Dong C, Qiu Q, Li C. Integrating gut microbiota immaturity and disease-discriminatory taxa to diagnose the initiation and severity of shrimp disease. Environ Microbiol. 2017;19(4):1490–501. doi: 10.1111/1462-2920.13701 28205371

[37] Evariste L, Barret M, Mottier A, Mouchet F, Gauthier L, Pinelli E. Gut microbiota of aquatic organisms: A key endpoint for ecotoxicological studies. Environ Pollut. 2019;248:989–99. doi: 10.1016/j.envpol.2019.02.101 31091643

[38] Fan L, Li QX. Characteristics of intestinal microbiota in the Pacific white shrimp *Litopenaeus vannamei* differing growth performances in the marine cultured environment. Aquaculture. 2019;505:450–61. doi: 10.1016/j.aquaculture.2019.02.075

[39] Smits WK, Lyras D, Lacy DB, Wilcox MH, Kuijper EJ. Clostridium difficile infection. Nat Rev Dis Primers. 2016;2:16020. doi: 10.1038/nrdp.2016.20 27158839 PMC5453186

[40] De Simeis D, Serra S. Actinomycetes: A never-ending source of bioactive compounds—an overview on antibiotics production. Antibiotics (Basel). 2021;10(5):483. doi: 10.3390/antibiotics10050483 33922100 PMC8143475

[41] Yu Q, Xie J, Huang M, Chen C, Qian D, Qin JG, et al. Growth and health responses to a long-term pH stress in Pacific white shrimp *Litopenaeus vannamei*. Aquac Rep. 2020;16:100280. doi: 10.1016/j.aqrep.2020.100280

[42] Falkowski PG, Fenchel T, Delong EF. The microbial engines that drive Earth’s biogeochemical cycles. Science. 2008;320(5879):1034–9. doi: 10.1126/science.1153213 18497287

[43] Grote J, Schott T, Bruckner CG, Glöckner FO, Jost G, Teeling H, et al. Genome and physiology of a model Epsilonproteobacterium responsible for sulfide detoxification in marine oxygen depletion zones. Proc Natl Acad Sci U S A. 2012;109(2):506–10. doi: 10.1073/pnas.1111262109 22203982 PMC3258601

