# Supplementary material for: White Feces Syndrome is closely linked to impaired antioxidant capacity, the aquaculture environment, and microbial dysbiosis in Litopenaeus vannamei reared in small-pond culture system
Source: PLoS One. 2026 Oct 22;21(10):e0357891. doi: 10.1371/journal.pone.0357891 (PMC13690251; doi:10.1371/journal.pone.0357891)
Supplement: Supplementary file 3 [file pone.0357891.s003.docx]

Table S1. List of abbreviations used in this manuscript.

| Abbreviation | Full term |
| --- | --- |
| WFS | White feces syndrome |
| CON | Control group |
| T-AOC | Total antioxidant capacity |
| SOD | Superoxide dismutase |
| GSH-Px | Glutathione peroxidase |
| MDA | Malondialdehyde |
| OTU | Operational taxonomic unit |
| ASV | Amplicon sequence variant |
| PCA | Principal component analysis |
| PCoA | Principal coordinate analysis |
| KEGG | Kyoto Encyclopedia of Genes and Genomes |
| PICRUSt | Phylogenetic Investigation of Communities by Reconstruction of Unobserved States |
| TN | Total nitrogen |
| TP | Total phosphorus |
| NO₂⁻-N | Nitrite nitrogen |
| NO₃⁻-N | Nitrate nitrogen |
| qPCR | Quantitative polymerase chain reaction |
