# Supplementary material for: White Feces Syndrome is closely linked to impaired antioxidant capacity, the aquaculture environment, and microbial dysbiosis in Litopenaeus vannamei reared in small-pond culture system
Source: PLoS One. 2026 Oct 22;21(10):e0357891. doi: 10.1371/journal.pone.0357891 (PMC13690251; doi:10.1371/journal.pone.0357891)
Supplement: Supplementary file 4 [file pone.0357891.s004.docx]

Table S2. Differences between intestinal and environmental microbial communities and their potential relationships with WFS development and shrimp performance.

| Microbial compartment | Indicator | Observed changes in WFS group | Possible effect |
| --- | --- | --- | --- |
| Intestinal microbiota | Alpha/Beta diversity | Community structure shifted | Microbial dysbiosis |
| Intestinal microbiota | Dominant taxa | Relative abundance changes | Altered intestinal function |
| Sediment microbiota | Community composition | Significant separation between groups | Environmental microbial disturbance |
| Sediment microbiota | Functional prediction | Altered metabolic pathways | Changes in nutrient cycling |
| Water environment | TN, TP, nitrogen compounds | Increased nutrient loading | Stress and disease susceptibility |
| Host physiology | SOD, GSH-Px, MDA, T-AOC | Antioxidant imbalance | Reduced resistance to disease |
