# Supplementary material for: White Feces Syndrome is closely linked to impaired antioxidant capacity, the aquaculture environment, and microbial dysbiosis in Litopenaeus vannamei reared in small-pond culture system
Source: PLoS One. 2026 Oct 22;21(10):e0357891. doi: 10.1371/journal.pone.0357891 (PMC13690251; doi:10.1371/journal.pone.0357891)
Supplement: Supplementary file 5 [file pone.0357891.s005.docx]

Table S3. Major diseases affecting shrimp aquaculture, their causes, symptoms, and current control strategies.

| Disease | Causative agent/type | Main symptoms | Prevention and control strategies |
| --- | --- | --- | --- |
| White spot disease (WSD) | Virus: White spot syndrome virus (WSSV) | White spots on carapace, lethargy, reduced feeding, high mortality | Biosecurity, pathogen-free larvae, water quality management, reducing stress |
| Acute hepatopancreatic necrosis disease (AHPND) | Bacteria: Vibrio parahaemolyticus carrying PirAB toxin genes | Hepatopancreatic atrophy, reduced growth, high mortality | Control of Vibrio populations, probiotics, improved pond management, biosecurity |
| Enterocytozoon hepatopenaei (EHP) infection | Microsporidian parasite: E. hepatopenaei | Growth retardation, uneven size, poor production performance | Pathogen-free seed selection, pond sanitation, preventing horizontal transmission |
| Infectious hypodermal and hematopoietic necrosis (IHHNV) | Virus: IHHNV | Growth deformities, runt-deformity syndrome | Screening broodstock and postlarvae, biosecurity |
| Yellow head disease (YHD) | Virus: Yellow head virus (YHV) | Yellow discoloration of cephalothorax, reduced feeding, mortality | Biosecurity, disease monitoring, environmental management |
| Vibriosis | Bacterial infection: various Vibrio spp. | Lethargy, tissue damage, mortality | Maintain water quality, reduce organic loading, probiotics, management practices |
| White feces syndrome (WFS) | Multifactorial syndrome associated with microbial imbalance, environmental stress, and possible pathogens | White fecal strings, abnormal intestine, reduced growth | Improve water quality, optimize feeding, microbial management, reduce environmental stress |
