# Supplementary material for: White Feces Syndrome is closely linked to impaired antioxidant capacity, the aquaculture environment, and microbial dysbiosis in Litopenaeus vannamei reared in small-pond culture system
Source: PLoS One. 2026 Oct 22;21(10):e0357891. doi: 10.1371/journal.pone.0357891 (PMC13690251; doi:10.1371/journal.pone.0357891)
Supplement: Supplementary file 6 [file pone.0357891.s007.docx]

Response to PLOS ONE Editorial Office

Response to Editorial Office Comments

We sincerely thank the Editorial Office for carefully checking our manuscript and providing valuable guidance. We have carefully revised the manuscript and addressed all formatting, funding, data availability, and submission requirements as follows.

Comment 1:

Please ensure that your manuscript meets PLOS ONE's style requirements, including those for file naming.

Response:

Thank you for your suggestion. We have carefully checked and revised the manuscript according to the PLOS ONE formatting guidelines, including manuscript structure, formatting style, file naming, and other presentation requirements. The revised manuscript has been prepared following the recommended PLOS ONE style templates.

Comment 2:

Funding information should not appear in the Acknowledgments section. Please remove funding-related text from the manuscript and update the Funding Statement.

Response:

Thank you for pointing this out. We have removed all funding-related information from the Acknowledgments section of the manuscript.

We would like to update the Funding Statement in the submission system as follows:

Updated Funding Statement:

This work was supported by the Modern Seed Industry Park for White Leg Shrimp of Guangdong Province (GDSCYY2022-005); Guangdong Provincial Department of Education Project (2022KTSCX052); Zhanjiang Science and Technology Bureau Marine Young Talents Project (2022E05007); and Guangdong Ocean University Doctoral Research Initiation Project (060302022008).

We kindly request the Editorial Office to update the Funding Statement in the online submission system accordingly.

Comment 3:

Please state the role of the funders in the study.

Response:

Thank you for this comment. The funding organizations provided financial support only and had no involvement in the design of the study, data collection and analysis, decision to publish, or preparation of the manuscript.

The updated Role of Funder statement is:

The funders had no role in study design, data collection and analysis, decision to publish, or preparation of the manuscript.

We kindly request the Editorial Office to update this statement in the online submission system.

Comment 4:

Authors are affiliated with the funding organization. Please confirm whether the funder had any role through the participating authors.

Response:

Thank you for highlighting this issue. We confirm that although some authors are affiliated with the institution receiving the funding support, the funders did not participate in the study design, data collection, data analysis, interpretation of results, decision to publish, or preparation of the manuscript.

We agree with the following statement:

The funder provided support in the form of research funding/material support for the authors [insert author initials if required], but did not have any additional role in the study design, data collection and analysis, decision to publish, or preparation of the manuscript. The specific contributions of these authors are described in the Author Contributions section.

The Author Contributions section has also been reviewed and revised to accurately reflect each author's role.

Comment 5:

The data repository does not meet PLOS standards. Please upload the minimal data set to an acceptable repository.

Response:

Thank you for this important comment. We have deposited the minimal data set required to reproduce the findings of this study in an appropriate public repository that meets PLOS ONE's data availability requirements.

The updated Data Availability Statement is:

All relevant data underlying the findings of this study are available from the [repository name] repository at [DOI/accession number/link].

(Please replace the bracketed information after uploading the data.)

Comment 6:

Please ensure that the corresponding author's ORCID iD is validated.

Response:

Thank you for your reminder. The corresponding author's ORCID iD has been added and validated in Editorial Manager.

Comment 7:

Please upload new copies of Figures 1–7 with improved resolution.

Response:

Thank you for the suggestion. We have regenerated and uploaded high-resolution versions of Figures 1–7 according to PLOS ONE figure preparation requirements. The figure quality and readability have been improved.

Comment 8:

Please include captions for Supporting Information files and update citations accordingly.

Response:

Thank you for this comment. We have added captions for all Supporting Information files at the end of the manuscript and carefully checked all in-text citations to ensure consistency with the Supporting Information files.

Comment 9:

Please evaluate suggested references from reviewers.

Response:

Thank you for this suggestion. We have carefully evaluated all references suggested by reviewers and included only those that are directly relevant to the scope and findings of our study. Additional references were added where scientifically appropriate, while unrelated references were not incorporated.

We sincerely appreciate the Editorial Office's careful review and helpful suggestions. We have revised the manuscript and submission information accordingly and hope that the revised version now meets all PLOS ONE requirements.

Reviewer #1: There are no recommendations / limitations

Response: Thank you. A paragraph describing the limitations of this study has now been added to the Discussion section.

LN/39---extensive or intensive and what are/are the differences

Response:

Thank you for this valuable comment. We agree that the original manuscript did not clearly define the aquaculture production system. We have revised the Introduction section to clarify the differences among extensive, semi-intensive, and intensive aquaculture systems based on stocking density, feed input, water exchange, and management intensity. In addition, we have specified that WFS is mainly reported in intensive shrimp culture systems and added explanations regarding the environmental characteristics of intensive farming systems.

Line 21-23

Conclusions are more than enough

Response:

There are no gross figures or images

Response: Thank you for this valuable suggestion. Unfortunately, gross photographs of shrimp were not collected during the original sampling period. We have therefore clarified the diagnostic criteria for WFS in the revised manuscript and acknowledged the lack of photographic documentation as a limitation of this study.

Some cited references with missing data—recheck

Response:

Thank you for this valuable suggestion. We carefully rechecked all references and corrected incomplete or inconsistent bibliographic information, including missing DOI numbers, journal information, volume, issue, and page numbers where applicable. The reference list has also been reformatted according to the journal guidelines to ensure consistency.

Huge number of references were detected—why

Response:

Thank you for this comment. We agree that excessive or unnecessary citations may reduce the clarity of the manuscript. We carefully reviewed the reference list and removed redundant or less relevant references. The remaining references were retained because they provide essential background information or directly support the interpretations related to WFS, shrimp physiology, intestinal microbiota, sediment microbial ecology, and aquaculture environmental management. We have also ensured that each citation is directly associated with the corresponding statement in the revised manuscript.

Which type of submission is this paper? abstract without backgrounds/methods/conclusion

Response:

Thank you for this comment. This manuscript was submitted as an Original Research Article. We agree that the previous abstract format did not clearly distinguish the background, methods, results, and conclusions. Therefore, we have revised the abstract into a structured format with four sections: Background, Methods, Results, and Conclusions, according to the journal requirements for research articles.

LN/76---2 different styles of writing references were detected—why—same style should be

Response:

Thank you for pointing out this issue. We carefully checked the entire manuscript and found inconsistencies in the citation style. All in-text citations have been revised and standardized according to the journal’s required format. The reference style is now consistent throughout the manuscript.

Abstract:

There are no highlights and should be written after the abstract not before

Response: Thank you for the suggestion. However, PLOS ONE does not require Highlights for Original Research Articles. Therefore, no Highlights have been added, and the manuscript has been prepared according to the journal's formatting requirements.

There is no graphical abstract

Response: Thank you for the suggestion. A graphical abstract is not required by the PLOS ONE submission guidelines. Therefore, we have prepared the manuscript in accordance with the journal's formatting requirements.

What is/are the creativity of this work?

Response: This manuscript is submitted as an Original Research Article in accordance with the PLOS ONE author guidelines.

LN/36-37-Avoid use of some words, already used with the main topic as keywords

Response:

Thank you for this helpful suggestion. We have carefully revised the Introduction section to reduce unnecessary repetition of keywords and improve the readability of the manuscript. Repeated use of terms already included as keywords, such as “white feces syndrome” and “intestinal microbiota,” has been minimized by replacing them with appropriate alternative expressions where possible.

Divide the abstract into the following items: --background/aims/methods/results and conclusion

Response: Thank you for this valuable suggestion. We appreciate the reviewer's recommendation. However, according to the PLOS ONE Instructions for Authors, Original Research Articles require an unstructured abstract rather than a structured abstract with separate headings. Therefore, we retained the journal-required format while revising the abstract to improve its clarity, readability, and presentation of the study objectives, methods, major findings, and conclusions.

Huge number of abbreviations were detected---it is better to create a separate table for

Response:

Thank you for this valuable suggestion. We agree that the large number of abbreviations used in the manuscript may affect readability. Therefore, we have added a separate table listing all abbreviations and their full definitions (Table S1). In addition, abbreviations have been checked throughout the manuscript to ensure that each term is defined at its first appearance.

LN/19---intensive shrimp aquaculture—what about stocking density/feeding program and aeriation

Response:

Thank you for this important comment. We agree that the criteria used to define the culture system as intensive should be clarified. We have added additional information regarding stocking density, feeding management, aeration practices, and water exchange in the revised manuscript. These characteristics support the classification of the experimental system as a small-pond intensive shrimp aquaculture system.

LN/26---dominant taxa---describe this

Response:

Thank you for this helpful suggestion. We agree that the term “dominant taxa” was not clearly defined in the original manuscript. We have revised the text to clarify that dominant taxa refer to microbial groups with the highest relative abundance within the microbial community. The definition has been added to the Introduction section.

Table the differences between intestinal microbial communities/environmental ones and its relation to the disease /growth performance and production of prawn

Response:

Thank you for this valuable suggestion. We agree that a comprehensive comparison between intestinal and environmental microbial communities would improve the interpretation of our findings. Therefore, we have added a new table summarizing the differences between intestinal and sediment microbial communities, their potential ecological functions, and their possible relationships with WFS development, shrimp health, and aquaculture production performance.

Add shrimp aquaculture/antioxidants/crustacean and oxidative stress to the keywords

Introduction

Response:

Thank you for this valuable suggestion. We have revised the keywords by adding “shrimp aquaculture”, “antioxidants”, “crustacean health”, and “oxidative stress” to better represent the major research themes of this study. In addition, the Introduction section has been expanded to provide more background information regarding antioxidant defense systems in crustaceans and the role of oxidative stress in shrimp health and disease susceptibility.

Add references after—LN/41/47/52/56/60/67/69/81/89 ---etc.

Response:

Thank you for this helpful suggestion. We carefully rechecked the Introduction section and added appropriate references after statements that required scientific support. Additional citations were incorporated at the indicated locations (Lines 41, 47, 52, 56, 60, 67, 69, 81, 89, and other relevant sections) to strengthen the background information and ensure that all important statements are properly supported by previous studies.

LN/39---extensive or intensive and what are/are the differences

Response:

Thank you for this important comment. We agree that the original manuscript did not clearly define the aquaculture production system. We have revised the Introduction section to clarify the differences between extensive and intensive shrimp farming systems based on stocking density, feed input, aeration, water exchange, and management intensity. We have also clarified that the present study was conducted in a small-pond intensive shrimp aquaculture system.

LN/44---disease outbreaks---tabulate all types of shrimp diseases according to the cause (for the producers and ordinary readers )/treatment also very important and control

Response:

Thank you for this valuable suggestion. We agree that a clearer overview of shrimp diseases would improve the accessibility of the manuscript for both researchers and aquaculture practitioners. We have added a summary table describing major shrimp diseases classified according to their causes (viral, bacterial, parasitic, and multifactorial), together with their main characteristics and current prevention/control strategies. This table provides additional background information while maintaining the focus of the present study on white feces syndrome.

LN/52---what about the cadmium pollution/fusarium ?

Response:

Thank you for this valuable comment. We agree that environmental contaminants and fungal pathogens may influence shrimp health and could potentially contribute to disease development. In the revised manuscript, we have expanded the Introduction to acknowledge the possible roles of heavy metal contamination (e.g., cadmium) and fungal pathogens (e.g., Fusarium spp.) in inducing oxidative stress, immune impairment, and microbial imbalance in aquatic animals. However, because the causal relationship between these factors and White Feces Syndrome (WFS) remains unclear, and because our study was designed to investigate the associations among antioxidant capacity, intestinal microbiota, sediment microbiota, and water quality, we have clarified that these factors require further targeted investigation in future studies.

The corresponding text has been added to the Introduction (Lines 69).

LN/59—different dysbiosis/symbiosis with example

Response:
Thank you for this helpful suggestion. We agree that the concepts of microbial symbiosis and dysbiosis should be better clarified for readers. In the revised Introduction, we have added a brief explanation describing the difference between a stable host–microbiota symbiotic relationship and microbial dysbiosis caused by environmental stress, infection, or nutritional imbalance. We have also provided examples showing that dysbiosis may involve the reduction of beneficial microbial groups and/or the enrichment of opportunistic microorganisms, which may impair intestinal homeostasis and increase disease susceptibility.

The corresponding text has been added to the Introduction (Lines 84).

LN/76---2 different styles of writing references were detected—why—same style should be

Response:

Thank you for pointing out this formatting inconsistency. We apologize for this oversight. During manuscript preparation, references were inserted using different citation formats, resulting in an unintended mixture of author–year and numbered citation styles. In the revised manuscript, we have carefully checked and reformatted all in-text citations and the reference list according to the journal's required style. All references are now presented consistently in the numbered citation format.

The entire manuscript and reference list have been carefully revised accordingly.

Introduction is very long and repeated

Response:

Thank you for this valuable suggestion. We agree that the previous version of the Introduction contained repetitive descriptions regarding the roles of WFS, intestinal microbiota, and environmental factors. In the revised manuscript, we have substantially shortened and reorganized the Introduction by removing redundant statements, merging overlapping sections, and improving the logical flow from WFS occurrence, microbial dysbiosis and oxidative stress, to the research gap and objectives of the present study.

The revised Introduction is now more concise and focused while maintaining the necessary scientific background.

Novelty should be more clarified

Response:

Thank you for this important suggestion. We agree that the novelty and significance of our study were not sufficiently emphasized in the previous version. In the revised manuscript, we have clarified the research gap and highlighted the unique aspects of this study in the Introduction and Discussion.

Specifically, we have emphasized that previous WFS studies have mainly focused on individual factors, such as specific pathogens or intestinal microbiota alterations. In contrast, this study integrates host antioxidant status, intestinal microbial communities, sediment microbiota, and water quality characteristics to investigate WFS from a host–microbiota–environment perspective in a practical small-pond culture system. This integrated approach provides new insights into the complex interactions associated with WFS occurrence.

The corresponding revisions have been added to the Introduction (Lines 116) and Discussion (Lines 451).

Aims should be more highlighted

Response:

Thank you for this valuable suggestion. We agree that the objectives of the study were not sufficiently highlighted in the previous version. In the revised manuscript, we have rewritten the final paragraph of the Introduction to clearly state the overall aim and specific objectives of this study.

The revised version now explicitly describes that this study aimed to investigate the associations among shrimp physiological responses, intestinal microbial dysbiosis, sediment microbial communities, and environmental factors during WFS outbreaks. The specific objectives, including the evaluation of antioxidant status, intestinal microbiota alterations, sediment microbial changes, and water quality characteristics, have also been clearly listed.

The corresponding revisions have been made in the Introduction (Lines 128).

Materials and Methods

There is no plan for the study area(s)

Response:

Thank you for this helpful suggestion. We agree that the description of the study area was insufficient in the previous version. Because WFS occurrence is closely associated with environmental conditions, detailed information regarding the sampling area and culture system is important for understanding the experimental context.

In the revised manuscript, we have added a new subsection entitled “Study area and shrimp culture system” in the Materials and Methods section. The revised text provides information on the geographical location, pond culture characteristics, management conditions, and selection of WFS-affected and healthy ponds.

The corresponding information has been added to the Materials and Methods section (Lines 144).

The whole experimental protocol was done according to whom!

Response:

Thank you for this important comment. We agree that the basis and ethical considerations of the experimental procedures were insufficiently described in the previous manuscript. In the revised version, we have added a statement describing the guidelines followed for shrimp handling, sampling, and experimental procedures.

Specifically, we have clarified that the sampling and experimental procedures were conducted according to the relevant guidelines for the care and use of aquatic animals and followed standard procedures for shrimp health investigations in aquaculture systems. The corresponding information has been added to the Materials and Methods section (Lines 174).

The most descriptive methodologies are without references –why?

Response:

Thank you for this valuable comment. We agree that several methodological descriptions in the previous manuscript lacked appropriate references. In the revised manuscript, we have carefully reviewed the Materials and Methods section and added relevant references for the established analytical procedures, including antioxidant capacity measurements, 16S rRNA gene sequencing analysis, and microbial functional prediction methods.

For methods that were performed strictly according to the manufacturer's instructions, we have clarified this information, while previously established protocols have now been supported by appropriate citations. These revisions improve the methodological transparency and reproducibility of the study.

There is no reference for the statistical analysis

Response:

Thank you for this valuable comment. We agree that the statistical methods used in this study were insufficiently supported by methodological references in the previous version. In the revised manuscript, we have added appropriate references for the statistical approaches, including PERMANOVA for microbial community comparison.

The Statistical Analysis section has been revised to include these methodological references, improving the transparency and reproducibility of the statistical procedures.

LN/112---clinical signs or symptoms and why

Response:

Thank you for this helpful comment. We agree that the terminology should be clarified. Because shrimp cannot report subjective symptoms, the term “clinical signs” is more appropriate than “clinical symptoms” for describing observable disease characteristics in aquatic animals. In the revised manuscript, we have replaced “clinical symptoms” with “clinical signs” and clarified that WFS-affected ponds were identified based on observable characteristics, including white fecal strings and reduced feeding activity.

We have also clarified that these clinical signs were used for field classification of WFS-associated ponds rather than definitive diagnosis, as WFS is considered a multifactorial disorder without a single confirmed causative agent.

The diagnosis of the disease depends upon what (case history/clinical signs /PM/pathology/isolation and identification---etc.??

Response:

Thank you for this important comment. We agree that the diagnostic criteria for WFS should be clearly described. In this study, the identification of WFS-associated ponds was based on farm history and typical clinical signs observed during routine monitoring, including white fecal strings, reduced feeding activity, and poor growth performance. Post-mortem examination and pathogen isolation/identification were not used as diagnostic criteria because this study aimed to investigate the associations among shrimp physiological status, microbial communities, and environmental factors in naturally occurring WFS conditions rather than identify a single causative agent.

We have clarified the WFS identification criteria in the Materials and Methods section and added a limitation regarding the absence of comprehensive pathogen screening in the revised Discussion.

LN/197---more details are requested

Response:

Thank you for this helpful suggestion. We agree that the description of statistical analyses was insufficiently detailed in the previous version. In the revised manuscript, we have expanded the Statistical Analysis section by clarifying the statistical software, assumptions testing, microbial diversity analyses, PERMANOVA parameters, correlation analysis procedures, and multiple comparison correction methods. These revisions improve the transparency and reproducibility of the statistical analysis.

Very long and rewrite it again

Response:

Thank you for this important suggestion. We agree that the Introduction was overly lengthy and contained some repetitive descriptions in the previous version. In the revised manuscript, we have substantially rewritten and shortened the Introduction by removing redundant background information, merging overlapping concepts, and improving the logical flow from WFS occurrence, microbial dysbiosis and oxidative stress, to the research gap and objectives. The revised Introduction is more concise and focused while maintaining the necessary scientific background and highlighting the novelty and aims of the study.

Results

What about the clinical signs of the investigated fishes

Response:

Thank you for this important suggestion. We agree that the clinical characteristics of WFS-associated shrimp should be clearly described. In the revised manuscript, we have added a new subsection entitled “3.1 Clinical observations” in the Results section, describing the major observable signs, including white fecal strings, reduced feeding activity, and sluggish swimming behavior. We have also clarified in the Materials and Methods section that WFS-associated ponds were classified based on farm history and typical clinical signs rather than definitive pathogen confirmation

What about the PM changes(cuticle/carapace ectoderm/mesoderm /abdominal muscles—etc. ----gross figures are mandatory

Response: Thank you for this valuable suggestion. We agree that gross photographs would provide additional visual information. However, this study was designed primarily to investigate antioxidant status, microbial communities, and environmental factors associated with WFS. Gross pathological images were not recorded during sampling. We have acknowledged this limitation in the revised Discussion and recommend that future studies include gross pathological documentation.

Are there any recorded mortalities and pathogenesis are important

Response:

Thank you for this important comment. We agree that mortality information and disease pathogenesis are important aspects for understanding WFS. In this study, no systematic mortality data were available because the investigation was conducted under naturally occurring field conditions rather than a controlled disease challenge experiment. The investigated ponds mainly showed sublethal clinical signs, including white fecal strings, reduced feeding activity, and sluggish swimming behavior.

We have clarified this point in the revised manuscript. In addition, we have expanded the Discussion to emphasize that WFS is considered a multifactorial disorder involving interactions among host physiological status, microbial dysbiosis, and environmental factors, while the exact pathogenesis remains to be further elucidated.

What about the pathological scores for confirmation and groups comparison

Response:

Thank you for this important suggestion. We agree that histopathological evaluation would provide additional evidence for confirming tissue-level alterations associated with WFS. However, histopathological scoring was not performed in the present study because the primary objective was to investigate the associations among antioxidant capacity, intestinal microbiota, sediment microbiota, and environmental factors under naturally occurring WFS conditions.

In the revised manuscript, we have clarified that WFS classification was based on farm history and typical clinical signs rather than histopathological confirmation. We have also added this point as a limitation and suggested that future studies combining histopathological scoring with pathogen detection and longitudinal monitoring will provide a more comprehensive understanding of WFS pathogenesis.

Why don’t you do a histological section for more confirmation

Response:

Thank you for this valuable suggestion. We agree that histological examination would provide additional evidence for confirming tissue-level pathological changes associated with WFS. However, histological analysis was not included in the current study because the primary objective was to investigate the associations among antioxidant capacity, intestinal microbial dysbiosis, sediment microbiota, and environmental factors under naturally occurring WFS conditions.

In this study, WFS-associated ponds were identified based on farm history and typical clinical signs, including white fecal strings and reduced feeding activity. We acknowledge that the absence of histopathological confirmation is a limitation of the present study. Therefore, we have added this point to the Discussion and suggested that future studies integrating histological examination, pathogen screening, and longitudinal monitoring are needed to further elucidate WFS pathogenesis.

WSDS---showed between bacteria/viral/PH—etc.

Response:

Thank you for this valuable suggestion. We agree that shrimp diseases should be clearly classified according to their etiological categories. In the revised manuscript, we have added a brief description of major shrimp diseases, including viral diseases (e.g., white spot disease caused by WSSV), bacterial diseases (e.g., AHPND and vibriosis), parasitic diseases (e.g., EHP infection), and multifactorial disorders such as WFS. We have also added a supplementary table summarizing the causative agents, clinical signs, and current control strategies of major shrimp diseases.

Discussion

LN/280---start from---in the present study ---etc.

Response:

Thank you for this suggestion. We agree that the final paragraph of the Introduction was too directly started with “In the present study” and did not sufficiently emphasize the scientific rationale and knowledge gaps. In the revised manuscript, we have rewritten this section by first highlighting the unresolved questions regarding the interactions among host physiological status, microbial dysbiosis, and environmental factors, followed by a clearer description of the study objectives.

The revised paragraph now better emphasizes the novelty and aims of the present study (Lines 370).

You should discuss your results obtained with the previous investigator’s results

Response:

Thank you for this valuable suggestion. We agree that the previous version of the Discussion focused mainly on the interpretation of our own findings and did not sufficiently compare them with previous studies. In the revised manuscript, we have expanded the Discussion by incorporating more comparisons with previous investigations regarding WFS-associated microbial dysbiosis, oxidative stress responses, and environmental microbial changes.

We have also discussed similarities and differences between our findings and previous reports and provided possible explanations based on differences in experimental conditions, shrimp culture systems, and disease complexity. These revisions strengthen the scientific context and highlight the contribution of the present study.

Repeated introduction

Too long

Rewrite it again

Response:

Thank you for this important suggestion. We agree that the previous version of the Introduction was overly lengthy and contained repeated descriptions of shrimp aquaculture background, WFS characteristics, and microbial dysbiosis. In the revised manuscript, we have completely rewritten the Introduction by removing redundant information, improving the logical flow, and focusing on the key scientific questions related to WFS.

Conclusion

More than enough

3-4 sentences are considered

Rewrite it again

Response: Thank you. The Conclusion has been substantially shortened to emphasize only the principal findings and their implications.

References

Some cited references need to be more updated

Response:

Thank you for this valuable suggestion. We agree that some references in the previous manuscript were relatively old and did not fully reflect recent advances in shrimp disease research and microbiome studies. In the revised manuscript, we have carefully re-evaluated the cited literature and updated several references, particularly those related to White Feces Syndrome, shrimp intestinal microbiota, microbial dysbiosis, oxidative stress responses, and aquaculture environmental interactions.

Several recent studies published within the last five years have been added to provide a more up-to-date scientific background and discussion. Meanwhile, classical references describing fundamental concepts and established methodologies have been retained where appropriate.

Some cited references with missing data—recheck

Response:

Thank you for this careful observation. We have thoroughly checked and revised the reference list to ensure that all cited references contain complete bibliographic information. Missing information, including author names, publication years, journal details, volume/pages, and DOI information, has been corrected where necessary. We have also standardized the reference format according to the journal guidelines.

As volume/issue/pages/number—all available—so no need for the link(s)—apply for all

Response:

Thank you for this suggestion. We have carefully revised the reference list according to the journal format. Since the volume, issue, page numbers, or article numbers are already provided for all references, DOI links and other hyperlinks have been removed throughout the reference list. The formatting of all references has been standardized accordingly.

Some journal names were written abbreviated, while others were not –why? Same style should be –apply for all

Response: Thank you. All references have been carefully reformatted to ensure consistency in journal names, citation style, volume, issue, and page numbers according to the PLOS ONE reference format.

Huge number of references(42) were detected—why

Response: Thank you for the comment. The references were selected to provide a comprehensive overview of previous studies related to white feces syndrome, oxidative stress, gut microbiota, and aquaculture environments. Nevertheless, we have carefully reviewed the reference list, removed redundant citations, and updated several references with more recent publications where appropriate.

All references should be rewritten

Response:

Thank you for this important suggestion. We have thoroughly revised the entire reference list according to the journal guidelines. All references have been reformatted to ensure consistency in author names, publication year, article title, journal information, volume, issue, and page numbers/article numbers. Missing bibliographic information has been completed, inconsistent reference styles have been corrected, and unnecessary hyperlinks/DOI links have been removed as suggested.

In addition, several outdated references related to WFS, shrimp microbiota, oxidative stress, and aquaculture environmental interactions have been replaced or supplemented with more recent publications.

Write as Table(1):---------/Fig.(1):--------etc

Response:

Thank you for this suggestion. We have carefully revised the manuscript and standardized all references to tables and figures throughout the text. Tables and figures are now consistently cited as “Table 1”, “Table 2”, “Fig. 1”, “Fig. 2”, etc., according to the journal formatting requirements.

Reviewer #2: 1. Please clarify whether the four replicates represent independent pond replicates or subsamples from the same pond.

Response: We appreciate the reviewer’s comment. The four replicates represent independent pond replicates rather than subsamples from the same pond. In the original manuscript, the description of the sampling site was insufficient and may have caused confusion. We have revised the Materials and Methods section to clarify that shrimp samples were collected from four independent small-pond culture systems, and each pond was considered an independent biological replicate. (Methods 2.2)

2. Please supplement the information on whether EHP infection status and Vibrio abundance were detected, and thoroughly elaborate on the limitations of this study.

Response: We agree with the reviewer that EHP and Vibrio are important factors associated with WFS. Unfortunately, pathogen-specific detection was not included in the present study. We have added this information as a limitation and emphasized that our results represent associations rather than causal evidence. (Discussion)

3. Description of water sampling is unclear

The manuscript states that water samples were collected from “each small pond”, whereas the Materials and Methods section only describes one pond. Please clarify the number of ponds involved and the sampling strategy used for water quality analysis.

Response: We appreciate the reviewer’s careful observation. The phrase “each small pond” was inaccurate because only one culture pond was involved in this study. We have corrected this description and clarified the sampling strategy.(Methods 2.3)

4. Correlation analysis was described but no results were presented

The authors stated in section 2.8 that Spearman correlation analysis was performed to evaluate relationships among environmental factors, antioxidant parameters, and dominant microbial taxa. However, no corresponding results are presented. Please either provide these results or remove this analysis from the Methods section.

Response: We agree with the reviewer. Because of the limited biological replicates, correlation analysis may not provide robust biological interpretation. Therefore, this analysis has been removed from the revised manuscript. (Methods 2.8)

5. Beta diversity analysis requires statistical support

The manuscript presents PCA plots, but no statistical evidence is provided to support group separation. Please perform PERMANOVA analysis and provide R² and P values to confirm whether microbial communities differ significantly between groups.

Response: We appreciate this suggestion. PERMANOVA analysis based on Bray–Curtis distance was performed, and the R² and P values have been added to the Results section and Methods.(Methods 2.6)

6. Functional prediction analysis needs clarification

The workflow used for PICRUSt analysis is confusing. The manuscript states that ASVs generated by DADA2 were clustered into OTUs at 97% similarity before functional prediction. Please explain the rationale for this procedure and provide additional information regarding software versions and databases used.

Response: We agree that the previous description was unclear. The OTU clustering step was incorrectly described and has been removed. We have clarified that PICRUSt2 prediction was performed directly using ASV representative sequences and abundance tables generated by DADA2. (Methods 2.7)

7. Data availability statement does not comply with PLOS ONE requirements

The current statement “Data will be made available on request” is insufficient. Raw sequencing data should be deposited in a public repository such as NCBI SRA, and accession numbers should be provided.

Response:

Thank you for this important suggestion. The processed sequencing datasets and supporting data have been deposited in Mendeley Data. However, we acknowledge that raw sequencing reads require deposition in a specialized nucleotide sequence repository. Therefore, we are currently preparing the raw 16S rRNA sequencing data for submission to the NCBI SRA database, and the accession number will be provided in the revised manuscript.

8. Line 95: the sentence “Recently, June to October are the high incidence months for White Feces Syndrome” is grammatically awkward. Please revise this sentence.

Response: We apologize for this inaccurate description. The sentence has been corrected.

9. Section 3.2 contains ambiguous wording.

The sentence:

“the concentration of MDA was significantly lower than that in the diseased group”

is difficult to follow. Please rewrite this section for clarity.

Response: We apologize for this inaccurate description. The sentence has been corrected.

10. Discussion mainly repeats the results and lacks mechanistic interpretation.

Response: We appreciate the reviewer’s valuable suggestion. We agree that the previous Discussion mainly summarized the observed changes and did not sufficiently explain the potential mechanisms underlying the interaction among oxidative stress, microbial dysbiosis, and environmental factors. In the revised manuscript, we have expanded the Discussion by adding a mechanistic interpretation of the host–microbiota–environment interaction during WFS development. We also clarified that the observed relationships represent associations rather than causal mechanisms due to the observational nature of this study.

Reviewer #3:

Introduction

Line 46–54: The Introduction mentions Vibrio spp. and Enterocytozoon hepatopenaei (EHP), but the Methods do not state whether these pathogens were tested. Please clarify whether EHP, Vibrio spp., or other shrimp pathogens were examined.

Response:

Thank you for this important comment. We agree that pathogen confirmation is critical for defining infectious diseases. In this study, WFS diagnosis was conducted under commercial pond conditions and was based on typical clinical signs, including white fecal strings, reduced feeding activity, empty intestine, and abnormal swimming behavior. We have clarified in the revised manuscript that the samples represent WFS-associated shrimp rather than experimentally infected shrimp.

In addition, we have added a limitation statement indicating that molecular detection of EHP and quantitative analysis of Vibrio spp were not performed, and therefore the contribution of specific pathogens to the observed microbial and physiological changes requires further investigation. (Lines 148-152)

Lines 50-52: The characterization of WFS as a multifactorial condition may need reconsideration. WFS is fundamentally a pathogen-derived disease, similar to other classical infectious diseases. It is highly likely that the factors mentioned—such as environmental stressors, microbial imbalance, and opportunistic pathogens—are secondary consequences of the primary WFS infection rather than the primary underlying causes. I suggest revising this phrasing to reflect this distinction.

Response: Thank you for this valuable comment. We agree that our previous wording may have overemphasized the contribution of environmental factors and microbial imbalance to WFS occurrence. In the revised manuscript, we have modified the description to better acknowledge the potential importance of pathogen-associated processes in WFS development while recognizing that environmental stressors, host physiological status, and microbial alterations may influence disease progression and severity.

The revised text now avoids implying that these factors are primary causes and instead describes them as potential interacting factors associated with WFS pathogenesis.

Line 52: Scientific names should be consistently italicized throughout the manuscript.

Response:

Materials and Methods

Line 84–95: The research gap is too broad. Please clearly state what is new compared with previous WFS studies on gut microbiota, oxidative stress, and environmental microbiota.

Response:

Thank you for this valuable suggestion. We agree that the previous description of the research gap was too general. In the revised manuscript, we have rewritten this section to clearly distinguish our study from previous WFS investigations. Specifically, we have emphasized that previous studies mainly focused on individual aspects, such as potential pathogens, intestinal microbiota, or host responses, whereas the present study integrates antioxidant status, intestinal microbial communities, sediment microbiota, and environmental parameters to investigate WFS-associated changes from a host–microbiota–environment perspective.

The revised Introduction now more clearly highlights the novelty and scientific significance of the present study. (Lines 110-114)

Line 104: The statement that June to October is the high-incidence period for WFS needs a supporting reference.

Response:

Thank you for this valuable suggestion. We agree that the original statement was too general and required additional support. We have revised this sentence to clarify that June to October represents the high-risk culture period observed in the study region rather than a universally fixed period for WFS occurrence. We have also added relevant references discussing the association between warm-season aquaculture conditions, environmental fluctuations, and increased risk of shrimp disease outbreaks.

Lines 105-107: Could the authors please clarify the methods used to confirm the disease status of the shrimp (e.g., specific clinical signs, PCR diagnosis)?

Response:

Thank you for this important comment. We agree that the criteria used for identifying WFS-associated shrimp were not sufficiently described in the previous version. In the revised manuscript, we have added detailed information regarding the field-based identification criteria.

Specifically, shrimp classification was based on typical WFS-associated clinical signs, including white fecal strings, reduced feeding activity, poor growth performance, and abnormal intestinal appearance observed during farm investigation. No pathogen-specific PCR diagnosis was performed in this field survey. Therefore, we have revised the terminology throughout the manuscript from “WFS shrimp” or “WFS-infected shrimp” to “WFS-associated shrimp” to avoid overinterpretation.

We have also added this limitation to the Discussion and highlighted that future studies integrating pathogen screening, histopathological examination, and longitudinal monitoring are needed for further confirmation of WFS etiology.

Lines 108-110: Please provide clearer definitions for the terms "early culture stage" and "late stage" within the context of this study.

Response:

Thank you for this helpful suggestion. We agree that the terms “early culture stage” and “late stage” were not sufficiently defined in the previous manuscript. In the revised version, we have clarified these terms according to the shrimp culture cycle investigated in this study. Specifically, the early culture stage refers to the initial period after stocking, whereas the late culture stage refers to the later period when shrimp biomass increases and environmental stress conditions become more pronounced.

The corresponding descriptions have been revised in the Introduction (Lines 157–159).

Line 105–113: WFS classification appears to be based only on clinical signs. Please define the diagnostic criteria.

Response:

Thank you for this important comment. We agree that the criteria used for WFS classification were insufficiently described in the previous manuscript. In the revised manuscript, we have added a detailed description of the diagnostic criteria used for identifying WFS-associated shrimp.

Specifically, shrimp classification was based on the presence of characteristic WFS-associated clinical signs, including white fecal strings, reduced feeding activity, sluggish swimming behavior, and abnormal intestinal appearance. Since this study was conducted under field conditions, pathogen-specific confirmation and experimental infection were not performed. Therefore, we have revised the terminology throughout the manuscript from “WFS shrimp” or “WFS-infected shrimp” to “WFS-associated shrimp” to accurately reflect the observational nature of this study.

Lines 111-112: It would be helpful to describe the typical clinical symptoms of WFS observed in this study. Furthermore, please explain the criteria used to identify "healthy" shrimp collected from a pond where diseased shrimp were present. Additionally, consider explaining why specific pathogens such as EHP or Vibrio were not screened or detected.

Response:

Thank you for this constructive comment. We agree that the criteria for identifying WFS-associated and healthy shrimp required further clarification. In the revised manuscript, we have added detailed descriptions of the clinical signs observed in this study, including floating white fecal strings, reduced feeding activity, sluggish swimming behavior, and intestinal abnormalities.

We have also clarified the criteria used for control shrimp selection. Healthy shrimp were defined as shrimp without visible WFS-associated clinical signs, including normal feeding behavior, active swimming, and no apparent intestinal abnormalities.

We acknowledge that pathogen-specific screening for EHP, Vibrio spp., and other potential pathogens was not performed in this field survey. This limitation has now been clearly stated in the revised Discussion, and we have clarified that the present study aimed to investigate the associations among host antioxidant status, microbial communities, and environmental factors rather than confirm specific pathogens responsible for WFS.

Lines 114-118: The description of the replicates is somewhat limited. Please provide a brief explanation of why different numbers of shrimp were used per group.

Response: We clarified that the four ponds represented four independent biological replicates and that pooled shrimp samples were technical composites representing each pond. Statistical analyses were conducted using pond-level replicates. (Lines 165-168)

Line 121–122: Sediment sampling is poorly described. Please provide key details such as sampling location, depth, amount, and number of subsamples, because sediment microbiota is a main part of this study.

Response:

Thank you for this valuable suggestion. We agree that the description of sediment sampling was insufficient in the previous manuscript. In the revised version, we have added detailed information regarding sediment sampling procedures, including sampling locations, sediment depth, sample amount, and the number of subsamples collected from each pond.

Specifically, sediment samples were collected from the upper sediment layer (0–5 cm) using sterile equipment. Multiple subsamples were collected from each pond and combined to obtain a representative sediment sample for microbiota analysis. The detailed sampling procedure has been added to the Materials and Methods section. (Lines 184)

Line 140–159: The 16S sequencing method is described generally, but it is not clear whether intestinal and sediment samples were processed using the same DNA extraction and amplification protocol. Please describe the procedures separately or clearly state that the same protocol was used for both sample types.

Response:

Thank you for this important suggestion. We agree that the previous description did not clearly distinguish the processing procedures for intestinal and sediment samples. In the revised manuscript, we have clarified whether the same DNA extraction, PCR amplification, and sequencing protocols were applied to both sample types.

Specifically, we have added detailed information regarding sample-specific DNA extraction procedures and clarified that the subsequent 16S rRNA gene amplification and sequencing procedures were performed using the same protocol for intestinal and sediment samples.

Line 160–168: The transition from sediment sampling to 16S analysis is abrupt. Since sediment microbiota is one of the main results of the study, the authors should provide more detailed sediment-specific processing information.

Response:

Thank you for this valuable suggestion. We agree that the previous description of sediment processing was insufficient, considering that sediment microbiota represents an important component of this study. In the revised manuscript, we have added a detailed description of sediment-specific processing procedures, including sample storage, homogenization, DNA extraction, and subsequent 16S rRNA gene sequencing procedures.

We have also clarified the relationship between sediment sample processing and the general microbiome workflow to improve the continuity and reproducibility of the methodology.

Line 169–172 and 192–199: Statistical methods are inconsistent. The Methods mention Kruskal–Wallis with Dunn’s post hoc test, but later mention Mann–Whitney U test. Please clarify the actual test used.

Response: Thank you for identifying this inconsistency. We have revised the Statistical Analysis section to clearly describe the statistical methods used for different datasets. Specifically, parametric and non-parametric tests were selected according to data distribution, Mann–Whitney U tests were used for alpha diversity comparisons, and PERMANOVA based on Bray–Curtis distances was applied for beta diversity analysis. We have also clarified the use of FDR correction for multiple comparisons.

Results

Overall: As a general note, please consider aligning the structure and order of the Results section with the Materials and Methods. Additionally, whenever the term "significantly" is used in the text, please ensure that the corresponding p-value is reported.

Response:

Thank you for this valuable suggestion. We have carefully revised the Results section to ensure that its structure and order are consistent with the Materials and Methods. The revised Results section now follows the same sequence as the experimental procedures, including clinical observations, environmental parameters, antioxidant responses, intestinal microbiota, sediment microbiota, and correlation analyses.

In addition, we have reviewed the entire manuscript and added the corresponding P values whenever the term “significantly” was used to describe statistical differences.

Line 203–206: Nitrate and total nitrogen are described as significantly decreased, but exact values and p-values are not provided in the text. Please add quantitative values.

Response:

Thank you for this helpful suggestion. We agree that the original description of water quality changes was insufficient. In the revised manuscript, we have added the quantitative values (mean ± SD) and corresponding statistical significance levels for nitrate and total nitrogen concentrations in the Results section. The revised text now provides both the magnitude of differences and the associated *P* values.

Line 231–233: The statement that WFS samples showed higher dispersion needs statistical support.

Response:

Thank you for this important comment. We agree that the previous statement regarding higher dispersion was based only on the visual interpretation of the PCoA plot and lacked statistical support. Therefore, we have revised the text to avoid overinterpretation and changed “higher dispersion” to “a tendency toward greater variability in microbial community composition.” We have clarified that this observation was interpreted cautiously.

Line 234–246: Several taxa are described as significantly increased or decreased, but exact relative abundances and p-values are missing. Please provide quantitative data.

Response:

Thank you for this important suggestion. We have revised the manuscript to provide quantitative information for the differential taxa. The relative abundances and p-values of the major bacterial taxa have now been added. Specifically, Clostridiaceae increased from 0.04% in the CON group to 5.06% in the WFS group (p = 0.029), whereas Intrasporangiaceae decreased from 7.63% to 3.49% (p = 0.029). Tessaracoccus showed a decreasing trend (2.85% to 0.93%), but the difference was not statistically significant (p = 0.200). We have revised the text accordingly to avoid overstating non-significant differences. Line 422-430.

244–246: The term “species” is inappropriate for 16S amplicon data unless species-level classification was confirmed.

Response:

Thank you for this important comment. We agree that the term “species” may be inappropriate for 16S rRNA amplicon sequencing data because species-level assignments cannot always be reliably confirmed. Therefore, we have carefully revised the manuscript and replaced “species” with more appropriate terms, such as “taxa”, “bacterial taxa”, or “genera”, depending on the corresponding taxonomic level. We have also clarified the taxonomic descriptions throughout the manuscript to avoid overinterpretation of 16S-based classifications.

Line 244–246: A Venn diagram alone does not show statistical significance. Please avoid saying “significantly higher” unless a statistical test was performed.

Response:

Thank you for this important comment. We agree that a Venn diagram only describes the distribution and overlap of ASVs between groups and cannot demonstrate statistical significance. Therefore, we have removed the term “significantly higher” from the manuscript and replaced it with a descriptive statement indicating differences in the number of unique ASVs. We have also carefully checked the related descriptions to avoid implying statistical significance without appropriate statistical tests.

Line 253–277: The sediment microbial results are extensively discussed, but the Materials and Methods do not provide sufficient sediment sampling details. This weakens the reproducibility and interpretation of the sediment microbiota data.

Response:

Thank you for this valuable comment. We agree that insufficient description of sediment sampling procedures may limit the reproducibility and interpretation of sediment microbiota results. Therefore, we have added detailed information regarding sediment collection, sampling depth, replication, sample handling, and storage conditions in the Materials and Methods section (Lines 182–184). In addition, we have revised the discussion of sediment microbial results to avoid overinterpretation and clarify that the observed sediment microbial changes represent associations with WFS rather than direct causal relationships.

Line 265–277: “WFS-infected samples” is inappropriate for sediment samples. Please revise to “sediment samples from the WFS group.”

Response:

Thank you for this helpful comment. We agree that the term “WFS-infected samples” is inappropriate for sediment samples because WFS is a disease condition of shrimp rather than sediment. We have revised the terminology throughout the manuscript and replaced “WFS-infected samples” with “sediment samples from the WFS group” or “sediment samples collected from WFS-affected ponds,” as appropriate.

Line 279–288: The authors suggest that lower nitrate and total nitrogen were caused by reduced feed intake or impaired nutrient assimilation.

Response:

Thank you for this important comment. We agree that our previous wording implied a causal relationship between lower nitrate/total nitrogen concentrations and reduced feed intake or impaired nutrient assimilation, which was not directly demonstrated in this study. We have revised the relevant sentences to indicate that these factors may be associated with altered nitrogen dynamics rather than confirmed causes. We have also added a statement acknowledging that further studies involving direct measurements of feed intake and nitrogen metabolism are needed.

Line 296: Please review and correct the formatting of the reference cited here to match the journal's guidelines.

Response:

Thank you for pointing out this issue. We have carefully checked the reference cited at Line 296 and corrected its formatting according to the journal's reference style guidelines. We have also reviewed the formatting of all references throughout the manuscript to ensure consistency with the requirements of PLOS ONE.

Line 364–370: The limitation section should mention lack of pathogen confirmation, pooled sampling, small sample size, limited sediment sampling information, and possible sample independence issues.

Response: We sincerely thank Reviewer #3 for the constructive comments. We agree that clearer descriptions of WFS diagnosis, sampling design, sediment microbiota analysis, and statistical interpretation were required. Accordingly, we have revised the manuscript by:

(1) clarifying that WFS diagnosis was based on clinical manifestations and revising terminology from "infected shrimp" to "WFS-affected shrimp";

(2) emphasizing that four independent ponds served as biological replicates and explaining the rationale for pooled shrimp samples;

(3) adding detailed sediment sampling information;

(4) revising microbial taxonomic descriptions to avoid inappropriate use of "species";

(5) adding quantitative statistical information and revising causal interpretations;

(6) expanding the limitation section regarding pathogen confirmation, sample independence, and observational design.
