# Supplementary material for: White Feces Syndrome is closely linked to impaired antioxidant capacity, the aquaculture environment, and microbial dysbiosis in Litopenaeus vannamei reared in small-pond culture system
Source: PLoS One. 2026 Oct 22;21(10):e0357891. doi: 10.1371/journal.pone.0357891 (PMC13690251; doi:10.1371/journal.pone.0357891)
Supplement: Supplementary file 7 [file pone.0357891.s001.docx]

Fig.S1 Comparison of water quality between the CON group and the WFS group (n = 4).
